# β-Blockers and Mortality After Acute Myocardial Infarction in Patients Without Heart Failure or Ventricular Dysfunction

**DOI:** 10.1016/j.jacc.2017.03.578

**Published:** 2017-06-06

**Authors:** Tatendashe B. Dondo, Marlous Hall, Robert M. West, Tomas Jernberg, Bertil Lindahl, Hector Bueno, Nicolas Danchin, John E. Deanfield, Harry Hemingway, Keith A.A. Fox, Adam D. Timmis, Chris P. Gale

**Affiliations:** a Medical Research Council Bioinformatics Centre, Leeds Institute of Cardiovascular and Metabolic Medicine, University of Leeds, Leeds, United Kingdom; b Leeds Institute of Health Sciences, University of Leeds, Leeds, United Kingdom; c Department of Medical Epidemiology and Biostatistics, Karolinska Institutet, Department of Cardiology, Karolinska University Hospital, Stockholm, Sweden; d Uppsala Clinical Research Center and Department of Medical Sciences, Uppsala University, Uppsala, Sweden; e Centro Nacional de Investigaciones Cardiovasculares, Madrid, Spain; f Instituto de Investigación i+12 and Cardiology Department, Hospital Universitario 12 de Octubre, Madrid, Spain; g Facultad de Medicina, Universidad Complutense de Madrid, Madrid, Spain; h Department of Cardiology, Hôpital Européen Georges Pompidou, Paris, France; i Assistance Publique-Hôpitaux de Paris, Paris, France; j Université Paris-Descartes, Paris, France; k National Institute for Cardiovascular Outcomes Research, University College London, London, United Kingdom; l University College London, London, and Farr Institute of Health Informatics Research, London, United Kingdom; m Centre for Cardiovascular Science, University of Edinburgh, Edinburgh, United Kingdom; n The National Institute for Health Biomedical Research Unit, Bart’s Heart Centre, London, United Kingdom; o Department of Cardiology, York Teaching Hospital NHS Foundation Trust, York, United Kingdom

**Keywords:** average treatment effect, NSTEMI, preserved left ventricular systolic function, propensity score, STEMI, survival, AMI, acute myocardial infarction, ATE, average treatment effect, CI, confidence interval, HF, heart failure, LVSD, left ventricular systolic dysfunction, MINAP, Myocardial Ischaemia National Audit Project, NSTEMI, non–ST-segment elevation myocardial infarction, PCI, percutaneous coronary intervention, STEMI, ST-segment elevation myocardial infarction

## Abstract

**Background:**

For acute myocardial infarction (AMI) without heart failure (HF), it is unclear if β-blockers are associated with reduced mortality.

**Objectives:**

The goal of this study was to determine the association between β-blocker use and mortality in patients with AMI without HF or left ventricular systolic dysfunction (LVSD).

**Methods:**

This cohort study used national English and Welsh registry data from the Myocardial Ischaemia National Audit Project. A total of 179,810 survivors of hospitalization with AMI without HF or LVSD, between January 1, 2007, and June 30, 2013 (final follow-up: December 31, 2013), were assessed. Survival-time inverse probability weighting propensity scores and instrumental variable analyses were used to investigate the association between the use of β-blockers and 1-year mortality.

**Results:**

Of 91,895 patients with ST-segment elevation myocardial infarction and 87,915 patients with non–ST-segment elevation myocardial infarction, 88,542 (96.4%) and 81,933 (93.2%) received β-blockers, respectively. For the entire cohort, with >163,772 person-years of observation, there were 9,373 deaths (5.2%). Unadjusted 1-year mortality was lower for patients who received β-blockers compared with those who did not (4.9% vs. 11.2%; p < 0.001). However, after weighting and adjustment, there was no significant difference in mortality between those with and without β-blocker use (average treatment effect [ATE] coefficient: 0.07; 95% confidence interval [CI]: −0.60 to 0.75; p = 0.827). Findings were similar for ST-segment elevation myocardial infarction (ATE coefficient: 0.30; 95% CI: −0.98 to 1.58; p = 0.637) and non–ST-segment elevation myocardial infarction (ATE coefficient: −0.07; 95% CI: −0.68 to 0.54; p = 0.819).

**Conclusions:**

Among survivors of hospitalization with AMI who did not have HF or LVSD as recorded in the hospital, the use of β-blockers was not associated with a lower risk of death at any time point up to 1 year. (β-Blocker Use and Mortality in Hospital Survivors of Acute Myocardial Infarction Without Heart Failure; NCT02786654)

Historically, β-blockers have been the standard of care for patients with acute myocardial infarction (AMI). However, clinical uncertainty exists regarding their effectiveness in reducing mortality among patients with AMI who do not have heart failure (HF) or left ventricular systolic dysfunction (LVSD). For example, although there is sufficient evidence to support the use of β-blockers in patients with AMI and HF 1, 2, as well as in hospitalized patients who are hemodynamically stable 3, 4, there are no contemporary randomized data for survivors of AMI without HF or LVSD in relation to the use of β-blockers. As such, international guidelines differ in their recommendation regarding the use of β-blockers after AMI 5, 6, 7, 8.

Results from recent observational studies suggest no significant association between the use of β-blockers among patients with AMI who do not have HF or LVSD and clinical outcomes. A meta-analysis comprising 16,645 patients with preserved left ventricular ejection fraction and who received percutaneous coronary intervention (PCI) for AMI found that the use of β-blockers was not associated with improved survival (9). However, recent data for 2,679 patients with AMI without HF or LVSD recorded in the FAST-MI (French Registry on Acute ST-Elevation and Non-ST-Elevation Myocardial Infarction) study found that early β-blocker use was associated with reduced 30-day mortality, but their discontinuation at 1 year was not associated with higher 5-year mortality (10).

To the best of our knowledge, to date, there are no analyses of large-scale datasets that have investigated the impact of β-blockers on survival after AMI among patients without HF or LVSD. On one hand, discontinuing β-blockers in survivors of AMI who do not have HF may prevent unnecessary overtreatment and costs, and improve adherence to other medications. On the other hand, randomized evidence to date suggests that use of β-blockers after AMI reduces clinical events 3, 11. The goal of the present study, therefore, was to use the United Kingdom national heart attack register, known as MINAP (Myocardial Ischaemia National Audit Project), to investigate the impact of the use of β-blockers on all-cause mortality at 1 year for survivors of hospitalized AMI without HF or LVSD.

## Methods

The analyses were based on data from MINAP, a comprehensive registry of acute coronary syndrome hospitalizations started in 2000 and mandated by the United Kingdom’s Department of Health (12). Data were collected prospectively at each hospital, electronically encrypted, and transferred online to a central database. Each patient entry offered details of the patient journey, including the method and timing of admission, inpatient investigations, results and treatment, comorbidities, risk factors, and (if applicable) date of death from linkage to the United Kingdom’s Office for National Statistics. Ethical approval was not required under National Health Service research governance arrangements. The National Institute for Cardiovascular Outcomes Research, which includes the MINAP registry (Ref: NIGB: ECC 1-06 [d]/2011), has support under section 251 of the National Health Service Act 2006 to use patient information for medical research without consent.

**Figure 1:**
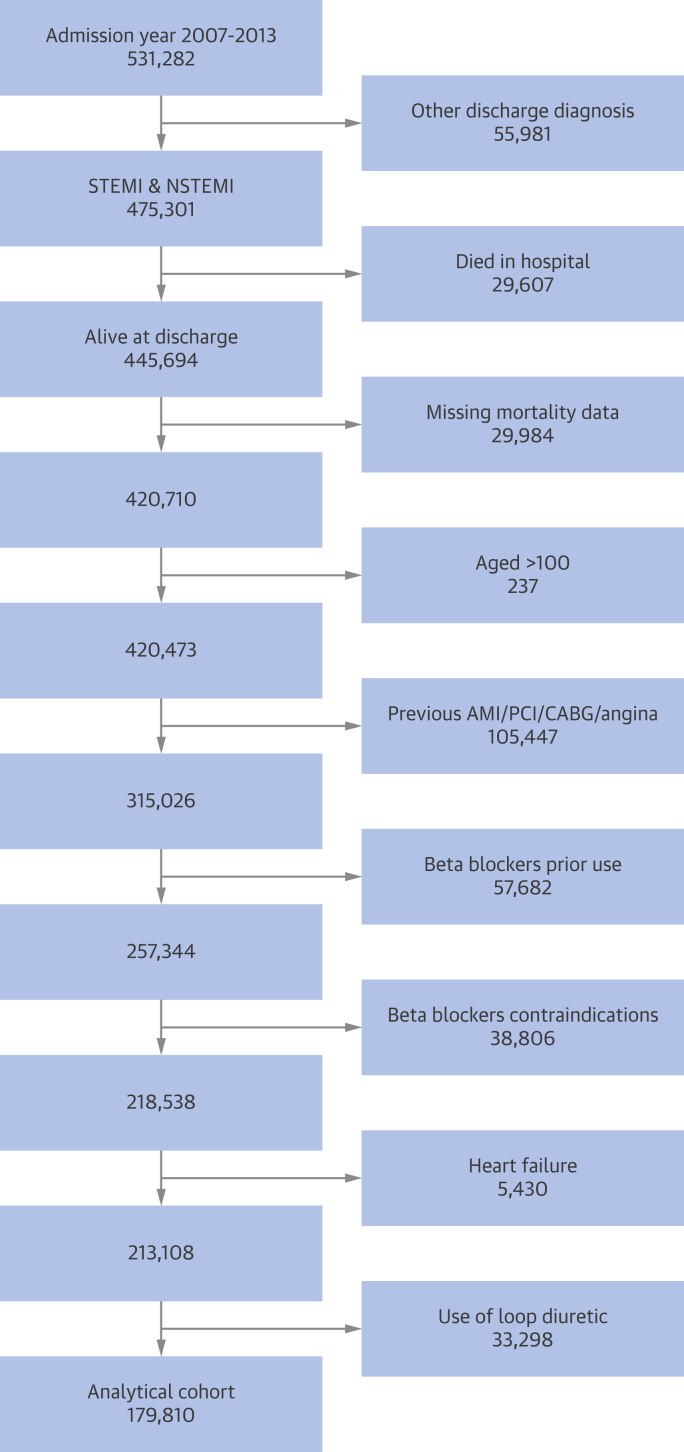
Analytical Cohort Derivation Flowchart Strengthening the Reporting of Observational Studies in Epidemiology diagram shows the derivation of the analytical cohort from the Myocardial Ischaemia National Audit Project dataset. AMI = acute myocardial infarction; CABG = coronary artery bypass graft; NSTEMI = non–ST-segment elevation myocardial infarction; PCI = percutaneous coronary intervention; STEMI = ST-segment elevation myocardial infarction.

The analytical cohort (N = 179,810) was drawn from 531,282 patients with AMI admitted to 1 of 247 hospitals between January 1, 2007, and June 30, 2013, with a final follow-up as of December 31, 2013 (Figure 1). Patients were eligible for the study if they were admitted from 2007 onward and discharged with a final diagnosis of ST-segment elevation myocardial infarction (STEMI) or non–ST-segment elevation myocardial infarction (NSTEMI). For patients with multiple admissions, the earliest record was used. We excluded 55,981 (10.5%) patients who had other final diagnoses; 29,607 (5.6%) who died in the hospital; 24,984 (4.7%) with missing mortality data; 237 (0.05%) >100 years of age; 105,447 (19.9%) who had previous AMI, angina, PCI, and/or coronary artery bypass graft surgery (and, therefore, may previously have received β-blockers); 57,682 (10.9%) who had a record of previous use of β-blockers; 38,806 (7.3%) with a contraindication to β-blockers; 5,430 (1.0%) who had a history of HF; and 33,298 (6.3%) who were prescribed a loop diuretic. For the present study, HF was defined as a history of HF, use of a loop diuretic on or during hospitalization, and/or a left ventricular ejection fraction <30% as recorded in the hospital. β-blocker use was determined according to whether eligible patients had received β-blockers at discharge from the hospital. The primary outcome was all-cause mortality at 1 year after hospitalization.

### Statistical analysis

Baseline characteristics according to treatment with β-blockers were described by using number and percentage for categorical data and mean ± SD or median and interquartile range for normally and non-normally distributed continuous data, respectively. Differences in characteristics were assessed by using chi-square tests, 2-sample Student *t* tests, and, for non-normally distributed data, the Mann-Whitney *U* test. Adjusted Kaplan-Meier curves were used to assess survival differences between patients who received β-blockers and those who did not.

Survival time inverse probability weighting propensity score analysis 13, 14 was used to evaluate the association between β-blocker use and mortality by estimating the average treatment effects (ATEs) and ATEs on the treated. Briefly, the method incorporated 2 models, the first of which was a treatment assignment model that estimated the propensity for β-blocker treatment assignment and was used to derive inverse probability weights. This model included 24 case-mix variables: sex; socioeconomic deprivation (Index of Multiple Deprivation score); year of hospital admission; cardiovascular risk factors (diabetes, hypercholesterolemia, hypertension, smoking status, and family history of coronary heart disease); chronic obstructive pulmonary disease; cerebrovascular disease; peripheral vascular disease; discharge medications (statins, aspirin, P2Y_12_ inhibitors, angiotensin-converting enzyme inhibitors/angiotensin receptor blockers); adjusted mini–Global Registry of Acute Coronary Events risk score variables (age, cardiac arrest, elevated enzyme, systolic blood pressure, heart rate at hospitalization, and creatinine level); and care by cardiologist. The second model was a survival model to determine the treatment effect using the inverse probability weights from model 1 to balance the covariate distribution between the treatment and control observations. To further mitigate from residual confounding in survival modeling, we adjusted for these covariates as well as cardiac rehabilitation. The second model was performed twice: first including only cases that were within 0.1 to 0.9 of the propensity score distribution to conduct a balanced analysis; and second including all cases regardless of their propensity score to check the robustness of the balanced analysis (the Online Appendix presents additional details).

Given that propensity scoring only adjusts for measured confounding, an instrumental variable analysis with hospital rates of prescription of guideline-indicated treatments (aspirin, P2Y_12_ inhibitors, β-blockers, statins, and angiotensin-converting enzyme inhibitors/angiotensin receptor blockers) as the instrument was used to further assess potential selection bias (the Online Appendix presents additional details). A Poisson regression analysis with an offset for the log survival time between discharge and final follow-up was used to provide a better approximation of the survival modeling framework. Analyses were undertaken for the overall AMI cohort and separately for cases of STEMI and NSTEMI, and effects were investigated at 1 month, 6 months, and 1 year.

To mitigate potential bias caused by missing data, we used multiple imputation by chained equations to create 10 datasets from 20 iterations; the resultant model estimates for each were combined by using Rubin’s rules (Online Appendix, Online Table 1). A complete case analysis was also conducted (Online Tables 2 and 3). Analyses were performed by using Stata MP64 version 14 (StataCorp, College Station, Texas) and R version 3.1.2 (R Foundation for Statistical Computing, Vienna, Austria). A p value <0.05 was considered statistically significant.

## Results

Of the 179,810 patients with AMI (91,895 with STEMI [51.1%]; 87,915 with NSTEMI [48.9%]) and no HF or LVSD who survived to discharge, 170,475 (94.8%) (88,542 with STEMI [96.4%]; 81,933 with NSTEMI [93.2%]) received β-blockers. There were significant differences in baseline characteristics between patients with and without β-blocker treatment (Table 1). In particular, patients who received β-blockers tended to be younger and male compared with those who did not receive β-blockers (mean age of 63.3 ± 13.4 years and 71.1% male vs. mean age of 68.6 ± 15.1 years and 61.7% male, respectively). Compared with patients who received β-blockers, those who did not were more frequently comorbid and of higher ischemic risk, including diabetes (15.4% vs. 11.6%), chronic renal failure (3.2% vs. 1.6%), asthma or chronic obstructive pulmonary disease (20.6% vs. 7.8%), cerebrovascular disease (7.0% vs. 3.8%), and with an intermediate or high Global Registry of Acute Coronary Events risk score (76.5% vs. 69.8%). Overall, the prescription of discharge medications, in-hospital procedures, and enrollment into cardiac rehabilitation was higher among those who received β-blockers.

**Table 1:** Baseline Characteristics

	β-Blockers at Time of Hospital Discharge∗		p Value	Missing
	Yes (n = 141,097)	No (n = 7,217)		
Age, yrs	63.3 ± 13.4	68.6 ± 15.1	<0.001	130 (0.07)
Male	100,774 (71.4)	4,441 (61.7)	<0.001	537 (0.3)
Deprivation (IMD)				
1 (least deprived)	24,615 (18.3)	1,379 (20.1)	<0.001	
2	26,677 (19.9)	1,381 (20.1)	0.639	
3	27,604 (20.6)	1,408 (20.5)	0.894	10,429 (5.8)
4	26,616 (19.8)	1,392 (20.3)	0.376	
5 (most deprived)	28,818 (21.5)	1,314 (19.2)	<0.001	
Year of admission				
2007	17,709 (12.6)	1,298 (18.0)	<0.001	
2008	19,369 (13.7)	1,230 (17.0)	<0.001	
2009	21,899 (15.5)	1,255 (17.4)	<0.001	
2010	23,720 (16.8)	1,107 (15.3)	0.001	
2011	24,925 (17.7)	1,115 (15.5)	<0.001	
2012	25,387 (18.0)	930 (12.9)	<0.001	0
2013	8,088 (5.8)	282 (3.9)	<0.001	
Cardiovascular history				
Cerebrovascular disease	4,835 (3.8)	457 (7.0)	<0.001	20,754 (11.5)
Peripheral vascular disease	2,365 (1.9)	210 (3.3)	<0.001	23,107 (12.9)
Cardiovascular risk factors				
Diabetes	15,785 (11.6)	1,076 (15.4)	<0.001	7,195 (4.0)
Chronic renal failure	1,953 (1.6)	208 (3.2)	<0.001	20,924 (11.6)
Hypercholesterolemia	33,788 (26.9)	1,710 (26.3)	0.305	21,838 (12.2)
Hypertension	47,040 (36.4)	2,814 (42.0)	<0.001	17,306 (9.6)
Current or ex-smoker	88,468 (65.7)	3,898 (58.5)	<0.001	10,654 (5.9)
Asthma or COPD	9,813 (7.8)	1,348 (20.6)	<0.001	21,752 (12.1)
Family history of CHD	44,056 (38.2)	1,699 (30.1)	<0.001	36,139 (20.1)
Presenting characteristics				
Systolic blood pressure, mm Hg	140.4 ± 27.1	138.7 ± 27.8	<0.001	35,001 (19.5)
Systolic blood pressure <90 mm Hg	2,824 (2.5)	200 (3.3)	<0.001	
Heart rate, beats/min	76.0 (66.0 to 89.0)	77.0 (64.0 to 90.0)	0.134	35,176 (19.6)
Heart rate >110 beats/min	6,070 (5.3)	416 (7.0)	0.196	
Creatinine, μmol/l	85.0 (72.0 to 99.0)	87.0 (74.0 to 106.0)	<0.001	32,003 (17.8)
Creatinine >200 μmol/l	1,159 (1.0)	166 (2.8)	<0.001	
Peak troponin, ng/ml†	4.8 (0.7–50.0)	1.7 (0.2–19.0)	<0.001	21,359 (11.9)
Peak troponin ≥0.06 ng/ml†	119,302 (95.5)	6,146 (93.0)	<0.001	
Cardiac arrest	5,449 (4.0)	178 (2.5)	<0.001	6,428 (3.6)
Electrocardiographic characteristics				
No acute changes	13,816 (10.4)	942 (14.5)	<0.001	
ST-segment elevation	69,888 (52.3)	2,364 (36.3)	<0.001	
Left bundle branch block	2,523 (1.9)	219 (3.4)	<0.001	10,360 (5.8)
ST-segment depression	15,063 (11.3)	867 (13.3)	<0.001	
T-wave changes only	20,150 (15.1)	1,171 (18.0)	<0.001	
Other acute abnormality	12,094 (9.1)	954 (14.7)	<0.001	
GRACE risk score				
Lowest (≤70)	11,358 (12.7)	496 (11.4)	0.011	
Low (71–87)	15,709 (17.5)	531 (12.2)	<0.001	68,471 (38.1)
Intermediate to high (>88)	62,676 (69.8)	3,342 (76.5)	<0.001	
Index event				
STEMI	75,697 (53.7)	2,539 (35.2)	<0.001	0
NSTEMI	65,400 (46.4)	4,678 (64.8)	<0.001	0
Medication at discharge‡				Missing§
Aspirin (n = 176,040‖)	137,509 (99.4)	5,929 (84.3)	<0.001	13,942 (7.9)
P2Y_12_ inhibitors (n = 173,967‖)	95,292 (97.3)	3,313 (72.9)	<0.001	60,385 (34.7)
ACE inhibitor/ARB (n = 165,575§)	126,812 (95.6)	4,222 (60.2)	<0.001	15,584 (9.2)
Statins (n = 176,979§)	137,402 (98.9)	5,479 (76.8)	<0.001	14,483 (8.2)
In-hospital procedures‡				
Coronary angiography (n = 173,473§)	91,738 (71.3)	4,024 (61.3)	<0.001	10,543 (6.1)
Coronary intervention (PCI/CABG) (n = 171,906§)	65,937 (58.7)	2,158 (41.9)	<0.001	33,905 (19.7)
Rehabilitation‡				
Enrollment in cardiac rehabilitation (n = 173,473§)	120,371 (94.7)	4,544 (76.9)	<0.001	16,505 (9.6)

Values are mean ± SD, n (%), or median (interquartile range).

ACE = angiotensin-converting enzyme; ARB = angiotensin receptor blocker; CABG = coronary artery bypass graft; CHD = coronary heart disease; COPD = chronic obstructive pulmonary disease; GRACE = Global Registry of Acute Coronary Events; IMD = Index of Multiple Deprivation; NSTEMI = non–ST-segment elevation myocardial infarction; PCI = percutaneous coronary intervention; STEMI = ST-segment elevation myocardial infarction.

∗ Total number of patients with missing information for β-blocker use at hospital discharge: 31,496.

† Peak troponin level was truncated at 50 ng/ml.

‡ Of the eligible patients for the care intervention.

§ Proportion missing of the eligible patients for the care intervention.

‖ Total eligible for care intervention.

For the entire cohort, with >163,772 person-years of observation (maximum 1-year follow-up), there were 9,373 deaths (5.2%). Unadjusted 1-year mortality was significantly lower for patients who received β-blockers compared with those who did not (4.9% vs. 11.2%; p < 0.001).

### Propensity score analyses

**Figure 2:**
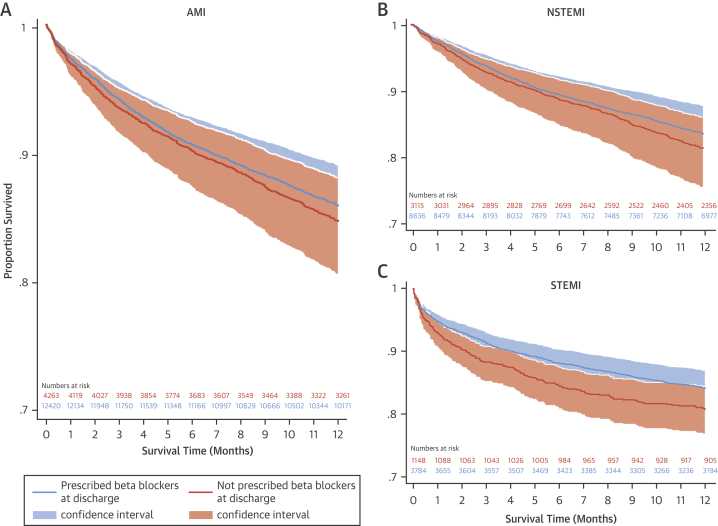
Adjusted Kaplan-Meier Survival Estimates (n = 16,683) In these adjusted survival curves according to prescription of β-blockers at discharge for the **(A)** AMI, **(B)** NSTEMI, and **(C)** STEMI groups, covariates and the inverse weighted propensity scores of receipt of care were adjusted for, and no statistical differences in survival were noted. Abbreviations as in Figure 1.

For the balanced propensity score analysis, 163,127 observations at the tails (i.e., outside the bounds of 0.1 to 0.9) of the estimated propensity score distribution were removed, leaving 16,683 patients (4,932 with STEMI [29.6%]; 11,751 with NSTEMI [70.4%]) for analysis. Overlap assumption assessment and balance checks were conducted and the results are summarized in Online Figures 1 to 5 and Online Tables 4 to 14, respectively. The assumption was not violated, and the covariates were balanced. The area under the curve for the propensity score model was 0.80 (Online Figure 6), which indicated a good discrimination for the model. After weighting and adjustment, there were no survival differences between patients with AMI and without HF or LVSD who received β-blockers and those who did not at any time point to 1 year (Figure 2, Online Figure 7). Specifically, at 1 month, 6 months, and 1 year after hospitalization with AMI, there was no significant difference in mortality when every patient in the analytical cohort used β-blockers compared with when no patients in the analytical cohort used β-blockers (ATE coefficient: 0.47; 95% confidence interval [CI]: −2.99 to 3.94 [p = 0.785]; ATE coefficient: 0.06; 95% CI: −0.35 to 0.46 [p = 0.768]; and ATE coefficient: 0.07; 95% CI: −0.60 to 0.75 [p = 0.827], respectively) (Table 2). There was also no significant treatment effect for the use of β-blockers at 1 month, 6 months, and 1 year for STEMI and NSTEMI.

**Table 2:** Effect of β-Blockers: Survival Time Inverse Probability Weighting Propensity Score Analysis

Trimmed Cohort Analysis					Full Analytical Cohort Analysis				
Follow-Up	ATE		ATET Only		Follow-Up	ATE		ATET Only	
	Coefficients∗ (95% CI)	p Value	Coefficients† (95% CI)	p Value		Coefficients∗ (95% CI)	p Value	Coefficients† (95% CI)	p Value
AMI (n = 16,683)					AMI (n = 179,810)				
1 month	0.47 (−2.99 to 3.94)	0.785	0.08 (−4.13 to 4.29)	0.971	1 month	0.04 (−1.54 to 1.61)	0.964	−0.11 (−1.78 to 1.56)	0.897
6 months	0.06 (−0.35 to 0.46)	0.768	−0.05 (−0.52 to 0.43)	0.849	6 months	0.0001 (−0.29 to 0.29)	0.999	−0.04 (−0.35 to 0.28)	0.820
1 yr	0.07 (−0.60 to 0.75)	0.827	0.02 (−0.80 to 0.85)	0.954	1 yr	0.47 (−0.13 to 1.08)	0.121	0.47 (−0.19 to 1.12)	0.159
STEMI (n = 4,932)					STEMI (n = 91,895)				
1 month	−0.14 (−5.89 to 5.61)	0.960	−0.50 (−7.06 to 6.06)	0.879	1 month	0.57 (−2.31 to 3.45)	0.693	0.54 (−2.20 to 3.28)	0.697
6 months	−0.15 (−0.97 to 0.67)	0.712	−0.28 (−1.27 to 0.72)	0.575	6 months	−0.33 (−0.87 to 0.20)	0.223	−0.40 (−0.95 to 0.15)	0.158
1 yr	0.30 (−0.98 to 1.58)	0.637	0.26 (−1.37 to 1.88)	0.748	1 yr	0.49 (−0.34 to 1.32)	0.246	0.49 (−0.36 to 1.36)	0.260
NSTEMI (n = 11,751)					NSTEMI (n = 87,915)				
1 month	0.12 (−3.34 to 3.58)	0.947	−0.72 (−4.95 to 3.52)	0.735	1 month	−0.16 (−3.62 to 3.31)	0.926	−0.45 (−4.22 to 3.33)	0.812
6 months	0.10 (−0.26 to 0.46)	0.565	0.02 (−0.38 to 0.42)	0.932	6 months	0.19 (−0.16 to 0.55)	0.286	0.18 (−0.20 to 0.56)	0.357
1 yr	−0.07 (−0.68 to 0.54)	0.819	−0.11 (−0.84 to 0.64)	0.777	1 yr	0.40 (−0.39 to 1.18)	0.314	0.39 (−0.48 to 1.26)	0.368

AMI = acute myocardial infarction; CI = confidence interval; other abbreviations as in Table 1.

∗ The average treatment effects (ATEs) represent the absolute difference in survival time (months, respective to the follow-up time category) between β-blocker treatment versus no treatment across the whole cohort (comparing survival times in a scenario in which all patients were treated versus survival times in a scenario in which no patients were treated).

† The average treatment effects on the treated (ATET) represent the absolute difference in survival time between β-blocker treatment versus no β-blocker treatment estimated only among those who were treated (comparing survival times for all β-blocker patients versus the potential survival time in the scenario that the treated patients did not receive β-blockers).

In the second propensity score analysis of 179,810 patients, and after weighting and adjustment, results were consistent with the balanced analysis. There was no significant association of β-blockers with survival at 1 month, 6 months, and 1 year for AMI overall or separately for STEMI and NSTEMI (Table 2).

### Instrumental variable analysis

The instrumental variable analysis of 179,810 cases found no significant difference in mortality at 1 month, 6 months, and 1 year for patients who did and did not receive β-blockers (coefficient: −0.003; 95% CI: −1.56 to 1.55 [p = 0.997]; coefficient: 0.18; 95% CI: −0.76 to 1.12 [p = 0.712]; and coefficient: 0.02; 95% CI: −0.64 to 0.68 [p = 0.953], respectively). This result was consistent across cases of STEMI and NSTEMI (Table 3). Validity of the instrumental variable was assessed, and the results are given in the Online Appendix and Online Table 15.

**Table 3:** Effect of β-Blockers: Instrumental Variable Analysis

	Treatment Effects	
	Coefficient∗ (95% CI)	p Value
AMI (n = 179,810)		
1 month	−0.003 (−1.56 to 1.55)	0.997
6 months	0.18 (−0.76 to 1.12)	0.712
1 yr	0.02 (−0.64 to 0.68)	0.953
STEMI (n = 91,895)		
1 month	−0.42 (−2.81 to 1.96)	0.725
6 months	0.32 (−2.54 to 3.18)	0.826
1 yr	0.03 (−1.82 to 1.87)	0.976
NSTEMI (n = 87,915)		
1 month	−0.57 (−1.64 to 0.49)	0.291
6 months	−0.34 (−0.91 to 0.22)	0.235
1 yr	−0.50 (−1.57 to 0.58)	0.365

Abbreviations as in Tables 1 and 2.

∗ Estimate represents the effect of β-blockers on survival for the respective follow-up time categories.

## Discussion

In this prospective, observational cohort study of the management and outcome of patients with acute coronary syndrome, using data for all hospitals in a single health care system, the use of β-blockers was not associated with a lower risk of death at up to 1 year among patients with AMI without HF or LVSD as identified during hospitalization (Central Illustration). Propensity score and instrumental variable analyses were used to provide insights into this important question from a large-scale, unselected patient population derived from the MINAP national registry.

**Central Illustration:**
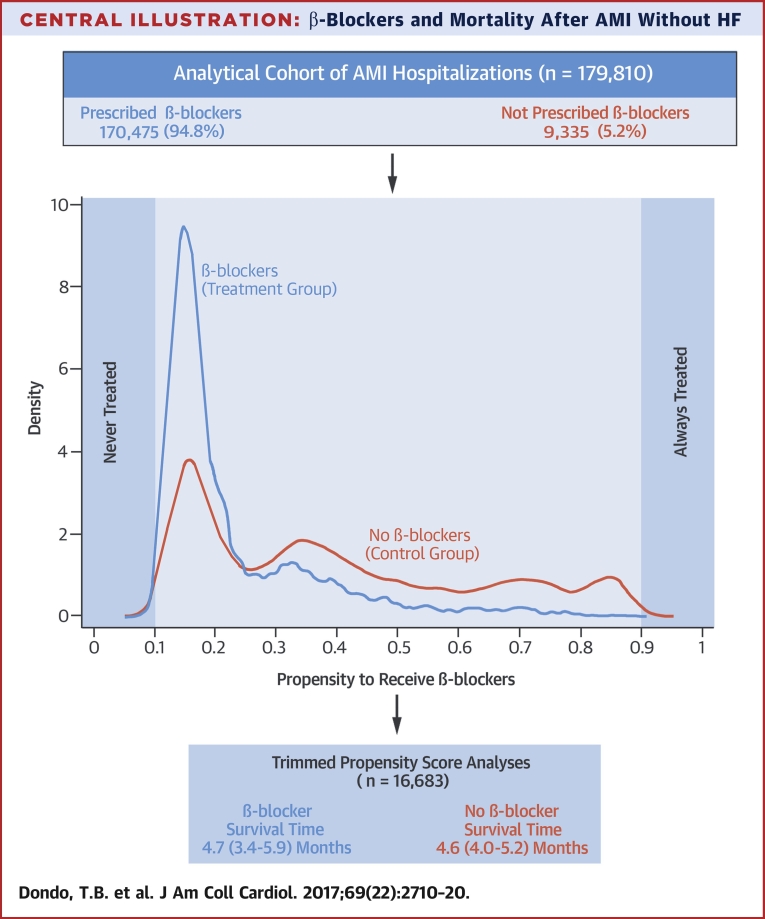
β-Blockers and Mortality After AMI Without HF In this study, patients experiencing an acute myocardial infarction (AMI) without heart failure (HF) or left ventricular systolic dysfunction were commonly prescribed β-blockers at hospital discharge (94.8%). However, in this nationwide observational study using propensity score analysis (1-year follow-up), the use of β-blockers was not associated with a significant difference in survival times after AMI.

Among nearly 17,000 (balanced propensity score analysis) and 180,000 (instrumental variable analysis) patients between 2007 and 2013 who were matched for demographic and clinical characteristics, the lack of association of β-blockers with survival was evident at 1 month, 6 months, and at 1 year after hospital discharge for STEMI and NSTEMI without HF or LVSD. These findings were in line with recommendations from recent guidelines for the management of acute coronary syndrome in patients with and without persistent ST-segment elevation 5, 15 and of clinical importance when the incidence of HF complicating AMI is in decline 16, 17.

However, international guidelines differ in their recommendations for the use of β-blockers after AMI, with U.S. guidelines recommending these drugs for all patients with AMI regardless of left ventricular ejection fraction or HF (Class I indication), whereas European guidelines have a Class IIa indication for those without LVSD or HF 5, 6, 7, 8. Many patients with AMI are prescribed β-blockers ad infinitum regardless of whether they have LVSD, HF, or neither (18). It is probable that this practice is, in part, supported by clinical uncertainty because evidence suggesting clinical benefit associated with the use of β-blockers in the context of AMI is varied, historical, extrapolated from nongeneralizable data, and unclear for AMI patients without HF. For example, although a meta-analysis of 31 studies reported an approximately one-quarter reduction in risk of death associated with β-blocker use, most of the included studies pre-dated the introduction of invasive coronary treatments (19). A meta-analysis of 10 observational studies across >40,000 patients suggested a lack of evidence to support the routine use of β-blockers in all patients with AMI who received PCI, but the effect was restricted to those with a reduced ejection fraction, NSTEMI, and those with low use of secondary prevention medications (9). Moreover, while β-blockers have been shown to be beneficial if given early after STEMI in patients who are hemodynamically stable, this effect is largely driven by a reduction in ventricular arrhythmias and reinfarction, and it was not known if there was a mortality advantage after 1 month of use among patients with STEMI or NSTEMI and who did not have HF or a preserved ejection fraction (4).

No randomized trials have tested the efficacy of β-blockers on long-term mortality among patients with AMI without HF or LVSD. Until now, the largest study, which comprised 6,758 propensity score–matched patients with AMI, found no reduction in mortality according to use of β-blockers (20). Notably, this study censored data in 2009 and did not investigate the impact of β-blockers on mortality among patients without HF or according to diagnosis of STEMI and NSTEMI. A smaller, but more recent study found that the discontinuation of β-blockers at 1 year was not associated with higher 5-year mortality (10). This finding is important because guidelines recommend that β-blockers be prescribed long term for patients after AMI who have HF, and it is uncertain as to whether β-blockers are beneficial for patients without HF but who have presented to the hospital with STEMI or NSTEMI.

In an era of coronary revascularization for AMI, whether it is primary PCI for acute STEMI or a risk-dependent early invasive strategy for NSTEMI, the likelihood of preserving more viable and therefore less arrhythmogenic myocardium is potentially greater than that of the noninterventional era. Arguably, in the absence of HF or LVSD, our study revealed that such patients who do not use β-blockers are at equal risk of death as those who do. Moreover, we found that the lack of effect of β-blockers on survival was evident for both STEMI and NSTEMI, and at early and later time points. Because β-blockers are not without potential harm, and given that many patients report side effects and that incremental numbers of medications are associated with reduced drug adherence (21), secondary prevention medications at hospital discharge for patients with AMI and without HF may not need to include β-blockers. Indeed, the European Society of Cardiology STEMI guidelines suggest that β-blockers be commenced in-hospital and continued long term after AMI but only with a Class IIa, Level of Evidence: B recommendation (6). For NSTEMI, a Class I, Level of Evidence: A recommendation is provided for the use of β-blockers but only in the context of HF (5). This recommendation contrasts with the current American College of Cardiology/American Heart Association guidelines, which recommend oral β-blockers as a Class I indication for all patients with AMI who do not have a contraindication 7, 8.

### Study limitations

Even though, to our knowledge, this study was the largest analysis to date (comprising >180,000 cases) of the effectiveness of β-blockers on mortality after AMI without HF or LVSD, our study was not without limitations. Only patients who survived the hospital stay were studied and, consequently, the role of in-hospital β-blockers was not investigated (e.g., for patients with early arrhythmias complicating AMI). The presence of HF or LVSD was only assessed by using data recorded during the hospital stay, and the risk of developing HF in the year after AMI, while declining, is not small 16, 17. In such circumstances, there is good evidence that β-blockers are beneficial and associated with lower mortality rates and better cardiovascular outcomes (22).

In addition, there was no information in the present study about rates of discontinuation, new prescriptions, or doses of β-blockers after hospital discharge. It is possible that nonpersistence with β-blockers explained the lack of impact on mortality or that patients who did not receive β-blockers at discharge received them later when reviewed in primary care. However, in the United Kingdom, patients receive a minimum of 1 month’s supply of medications at hospital discharge; only if patients in the treatment arm were nonadherent with their medications would this explain our study findings. Moreover, in the United Kingdom, β-blocker persistence is high after an AMI (18). Although the unadjusted analysis revealed a large difference in mortality rates between those who did and did not receive β-blockers, the difference was not observed after adjusting for confounders and selection bias using propensity score analysis. This finding likely reflects the fact that unadjusted analyses in observational data might be influenced by confounding (e.g., the use of other medical treatments) as well as selection bias. Notably, our study was a select and nonrandomized sample; in addition, although propensity scoring and instrumental variable analysis adjusted for confounding by indication, and further adjustments were made for many additional confounders in the survival models, residual confounding is probable. Nonetheless, our results are consistent with other nonrandomized data, albeit these studies used post hoc analyses to investigate the impact of β-blockers on mortality among AMI patients without HF or LVSD (10). Clearly, a randomized controlled trial is a necessary next step for the contemporary evaluation of β-blockers in AMI without HF or LVSD.

## Conclusions

Among patients who survived hospitalization in England and Wales with STEMI and NSTEMI without HF or LVSD, β-blocker use was not associated with lower all-cause mortality at any time point up to 1 year. This result adds to the increasing body of evidence that the routine prescription of β-blockers might not be indicated in patients with a normal ejection fraction or without HF after AMI.

Perspectives **COMPETENCY IN MEDICAL KNOWLEDGE:** Among hospital survivors of AMI without HF or LVSD, use of β-blockers was not associated with a lower risk of death at 1 year.

**TRANSLATIONAL OUTLOOK:** Clinical trials are needed to prospectively evaluate the efficacy of β-blockers in patients with AMI who do not have HF or LVSD.
