# Supplementary material for: β-Blockers and Mortality After Acute Myocardial Infarction in Patients Without Heart Failure or Ventricular Dysfunction
Source: J Am Coll Cardiol. 2017 Jun 6;69(22):2710–20. doi: 10.1016/j.jacc.2017.03.578 (PMC5457288; doi:10.1016/j.jacc.2017.03.578)
Supplement: Online Data [file mmc1.docx]

**SUPPLEMENTAL MATERIAL**

**Title: β blockers and mortality following acute myocardial infarction without heart failure or left ventricular systolic dysfunction**

**Authors**

Tatendashe B Dondo (MSc), Marlous Hall (PhD), Robert M West (DPhil), Tomas Jernberg (FRCP), Bertil Lindahl (FRCP), Hector Bueno (MD, PhD), Nicolas Danchin (MD), John E Deanfield (FRCP), Harry Hemingway (FFPH, FRCP), Keith AA Fox (FRCP), Adam D Timmis (FRCP), Chris P Gale (PhD, FRCP).

**Section 1: Multiple Imputation**

Multiple imputation by chained equations (MICE) ([1](#_ENREF_1)) was used to create 10 imputed datasets to mitigate potential bias caused by missing data for selected variables (Table 1). A default imputation strategy (missing data default imputed to “NO”) based on expert knowledge of the MINAP database was implemented for cardiovascular history, cardiovascular risk factors, and categorical treatment variables, where missing data is most likely to mean the patient did not have the condition or receive the treatment.([1](#_ENREF_1)) The imputation model specifications are defined in detail in Table 1. Predictive mean matching was used for continuous variables with nonlinear associations as in our previous work.([1](#_ENREF_1)) The complete case analysis results are summarised in Tables 2 and 3.

**Table 1.** Imputation specification

| **Variable** | **Variable Type** | **Missing (%)** | **Imputation method** |
| --- | --- | --- | --- |
| Cardiac arrest | Binary | 3.6 | Logistic regression |
| Uncensored peak troponin measurement in ng/ml | Continuous | 11.9 | Predictive mean matching |
| Age | Continuous | 0.07 | Predictive mean matching |
| Systolic blood pressure | Continuous | 19.5 | Predictive mean matching |
| Heart rate | Continuous | 19.6 | Predictive mean matching |
| Loop diuretic used | Binary | 17.4 | Logistic regression |
| Creatinine level | Continuous | 17.8 | Predictive mean matching |
| Ethnicity | Categorical |  | Polytomous regression |
| Sex | Binary | 0.3 | Logistic regression |
| Index of multiple deprivation score | Continuous | 5.8 | Predictive mean matching |
| Derived identification | Continuous | 0 | Predictor/ Auxiliary /Partially Observed |
| Arrival year | Continuous | 0 | Predictor/ Auxiliary /Partially Observed |
| Nelson-Aalen survival estimate | Continuous | 0 | Predictor/ Auxiliary /Partially Observed |
| Censoring indicator | Binary | 0 | Predictor/ Auxiliary /Partially Observed |
| Hypercholesterolaemia | Binary | 12.2 | Predictor/ Auxiliary and Default imputed |
| Previous hypertension | Binary | 9.6 | Predictor/ Auxiliary and Default imputed |
| Peripheral vascular disease | Binary | 12.9 | Predictor/ Auxiliary and Default imputed |
| Cerebrovascular disease | Binary | 11.5 | Predictor/ Auxiliary and Default imputed |
| Chronic obstructive pulmonary disease/Asthma | Binary | 12.1 | Predictor/ Auxiliary and Default imputed |
| Smoker ever | Binary | 5.9 | Logistic regression |
| Diabetes | Binary | 4.0 | Predictor/ Auxiliary and Default imputed |
| Family history of chronic heart disease | Binary | 20.1 | Predictor/ Auxiliary and Default imputed |
| Care by Cardiologist | Binary | 39.1 | Predictor/ Auxiliary and Default imputed |
| Chronic renal failure | Binary | 11.6 | Predictor/ Auxiliary and Default imputed |
| Electrocardiogram appearance | Categorical | 5.8 | Polytomous regression |
| Aspirin at discharge | Categorical | 7.9 | Polytomous regression |
| P2Y_12_ inhibitors at discharge | Categorical | 34.7 | Polytomous regression |
| ACEi/ARBs at discharge | Categorical | 9.2 | Polytomous regression |
| Statin at discharge | Categorical | 8.2 | Polytomous regression |
| Coronary angiography | Categorical | 6.1 | Polytomous regression |
| Enzyme elevation | Binary | 6.0 | Predictor/ Auxiliary variable |
| Admission diagnosis | Categorical | 0.02 | Predictor/ Auxiliary variable |
| Care by cardiologist | Binary | 8.4 | Logistic regression |
| Admitting consultant | Binary | 5.4 | Predictor/ Auxiliary variable |
| Final diagnosis | Binary | 0 | Predictor/ Auxiliary variable |
| Serum cholesterol | Continuous | 31.3 | Predictor/ Auxiliary variable |
| Admission method | Categorical | 66.9 | Predictor/ Auxiliary variable |
| Coronary intervention | Categorical | 19.7 | Polytomous regression |
| β blockers at discharge | Binary | 17.5 | Logistic regression |
| Cardiac rehabilitation | Categorical | 9.6 | Polytomous regression |

**Abbreviations:** ACE, angiotensin converting enzyme inhibitor; ARB, angiotensin receptor blocker.

**Table 2.** Effect of β blockers at discharge on all-cause mortality following AMI (survival-time inverse-probability weighting propensity score analysis) (trimmed complete case analysis)

| **Average treatment effects** | | | **Average treatment effects on the treated only** | | |
| --- | --- | --- | --- | --- | --- |
| **Follow-up** | **Coefficient^¥^ (95% CI)** | ***P*-value** | **Follow-up** | **Coefficient^¥^ (95% CI)** | ***P*-value** |
| **AMI** |  |  | **AMI** |  |  |
| One month | - | - | One month | - | - |
| Six months | -0.15 (-0.63 to 0.34) | 0.554 | Six months | -0.23 (-0.76 to 0.29) | 0.386 |
| One year | -0.32 (-1.23 to 0.60) | 0.495 | One year | -0.35 (-1.35 to 0.64) | 0.488 |
| **STEMI** |  |  | **STEMI** |  |  |
| One month | - | - | One month | - | - |
| Six months | 0.32 (-0.64 to 1.29) | 0.511 | Six months | 0.02 (-1.04 to 1.08) | 0.972 |
| One year | 0.15 (-1.96 to 2.26) | 0.887 | One year | -0.52 (-2.81 to 1.77) | 0.656 |
| **NSTEMI** |  |  | **NSTEMI** |  |  |
| One month | - | - | One month | - | - |
| Six months | -0.19 (-0.71 to 0.32) | 0.460 | Six months | -0.19 (-0.73 to 0.35) | 0.494 |
| One year | -0.53 (-1.58 to 0.53) | 0.327 | One year | -0.50 (-1.64 to 0.64) | 0.389 |

**Abbreviations**: AMI, acute myocardial infarction; ^¥^Estimate represents the effect of β blockers on survival for the respective follow-up time categories; NSTEMI, non ST-segment elevation myocardial infarction; STEMI, ST-segment elevation myocardial infarction,- model converge problems.

**Table 3.** Effect of β blockers at discharge on all-cause mortality following AMI (survival-time inverse-probability weighting propensity score analysis) (complete case analysis)

| **Average treatment effects** | | | **Average treatment effects on the treated only** | | |
| --- | --- | --- | --- | --- | --- |
| **Follow-up** | **Coefficient^¥^ (95% CI)** | ***P*-value** | **Follow-up** | **Coefficient^¥^ (95% CI)** | ***P*-value** |
| **AMI** |  |  | **AMI** |  |  |
| One month | 0.48 (-2.82 to 3.79) | 0.776 | One month | 0.24 (-3.26 to 3.73) | 0.895 |
| Six months | -0.08 (-0.63 to 0.47) | 0.782 | Six months | -0.13 (-0.71 to 0.45) | 0.666 |
| One year | 0.64 (-0.26 to 1.56) | 0.164 | One year | 0.70 (-0.27 to 1.66) | 0.156 |
| **STEMI** |  |  | **STEMI** |  |  |
| One month | -0.002 (-1.99 to 1.98) | 0.999 | One month | -0.10 (-2.13 to 1.93) | 0.924 |
| Six months | -0.68 (-1.67 to 0.29) | 0.168 | Six months | -0.73 (-1.74 to 0.28) | 0.155 |
| One year | 0.69 (-0.89 to 2.27) | 0.393 | One year | 0.68 (-0.94 to 2.31) | 0.411 |
| **NSTEMI** |  |  | **NSTEMI** |  |  |
| One month | - | - | One month | - | - |
| Six months | 0.42 (-0.17 to 1.01) | 0.166 | Six months | 0.44 (-0.19 to 1.08) | 0.169 |
| One year | 0.74 (-0.24 to 1.71) | 0.138 | One year | 0.86 (-0.18 to 1.90) | 0.104 |

**Abbreviations**: AMI, acute myocardial infarction; ^¥^Estimate represents the effect of β blockers on survival for the respective follow-up time categories; NSTEMI, non ST-segment elevation myocardial infarction; STEMI, ST-segment elevation myocardial infarction,- model converge problems.

**Section 2: Survival-time inverse-probability weighting propensity score analysis**

A non-parsimonious multivariable logistic regression model was used for the treatment model and a Weibull survival model for the time to event model. Both models were adjusted for patient demographics (sex, deprivation (index of multiple deprivation score), year of admission to hospital), cardiovascular risk factors (diabetes, hypercholesterolaemia, hypertension, smoking status, chronic obstructive pulmonary disease (COPD), family history of coronary heart disease), cardiovascular history (cerebrovascular disease, peripheral vascular disease), discharge medications (statins, aspirin, P2Y_12_ inhibitors, angiotensin converting enzyme inhibitors (ACEi)/angiotensin receptor blockers (ARB)), adjusted mini-GRACE risk score variables (age, cardiac arrest, elevated enzyme, systolic blood pressure and heart rate at hospitalisation and creatinine) and care by cardiologist. Cardiac rehabilitation was added only to the survival model because it is a post treatment variable and therefore cannot predict treatment assignment. In order to assess whether the weights constructed from the treatment assignment model balanced the covariates between treated and control individuals standardised differences and variance ratios of the raw data versus weighted data were calculated (a perfectly balanced covariate has a standardised difference of zero and variance ratio of one). A formal over-identification test for covariate balance was also used ([2](#_ENREF_2)). Violation of the overlap assumption was assessed using overlap plots and by summarising the estimated probabilities of treatment assignment. Observations with estimated propensity scores outside the pre-specified range 0.1 to 0.9 were discarded([3](#_ENREF_3)) for the first analysis, however, a second analysis was performed which included all observations regardless of their propensity score to assess the robustness of the results. Including the observations outside the bounds 0.1 to 0.9 would have resulted to the violation of the second assumption of the propensity modelling that assumes that for propensity score analysis to assume unconfoundedness, the estimated propensity scores for all observations should be greater than zero and less than one. Aspirin and ACEi/ARB at discharge were found to be poorly balanced, thus interaction terms of these variables with all of the other 24 model variables were added to the treatment assignment model.

The results of the assessment of the overlap assumption are shown in Figure 1-5, the minimum propensity score for each treatment level was sufficiently greater than zero and the maximum propensity score for each treatment level sufficiently less than 1 thus the assumption was not violated. Assessment was done across each of the ten imputed datasets individually as methods to pool propensity scores have not been defined, however the treatment effects were estimated based on pooled estimates from the imputed data. The balance check results are summarised in Tables 4-13 and most of the standardised differences and variance ratios for variables in the weighted data were close to zero and one, respectively. Balance checks were only performed for the main effects. The over-identification test further assessed whether the main effects as well as the interactions terms were balanced (Table 14). There was no evidence against the null hypothesis that the covariates were balanced thus the treatment assignment models were well specified. The diagnostic assessments suggest that weighting by the inverse probability of treatment created a sample in which the prevalence of binary baseline variables were similar between the treated and control subjects. The area under the curve for the propensity score model was 0.80 (Figure 6), which indicated a good discrimination for the model. Treatment effects were estimated as average treatment effects (ATE) and average treatment effects on the treated (ATET). The ATE coefficient represents the absolute difference in survival time estimated by calculating the survival times if all patients had received β blockers compared to the survival time if no patients received β blockers. Therefore, for example, a significant positive ATE coefficient of 1.00 would indicate that a patient could have survived 1.00 month more had they been treated with β blockers. The average treatment effects on the treated (ATET) coefficient represents the absolute difference in survival time only for those who were treated with β blockers and compares this to the survival time if those treated with β blockers were left untreated. The ATE and ATET were calculated using the following calculations:

For each subject, the effect of treatment is defined to the difference between two potential outcomes. This can be presented as Y*i* (1)-Y*i* (0), where Y*i* (0) and Y*i* (1) are the outcomes (survival time) under the control (untreated) and the treatment (treated) groups respectively. The ATE is the average of moving the entire population from treated to untreated, i.e. the E [Y*i* (1)-Y*i* (0)]. The average treatment effect on the treated (ATET) is the average effect of treatment on those subjects who ultimately received the treatment, i.e. the conditional expectation expressed as follows (E [Y*i* (1)-Y*i* (0)| Z=1], where Z=1 is for the treated patients only) .([4](#_ENREF_4)) Adjusted survival (Kaplan-Meier estimates) curves for the full analytical cohort (N= 179,810) were plotted and showed similar results as the trimmed analytical cohort (N= 16,683) analysis (Figure 7).

**Figure 1.** Overlap assumption assessment plots for imputation 1 and 2

**
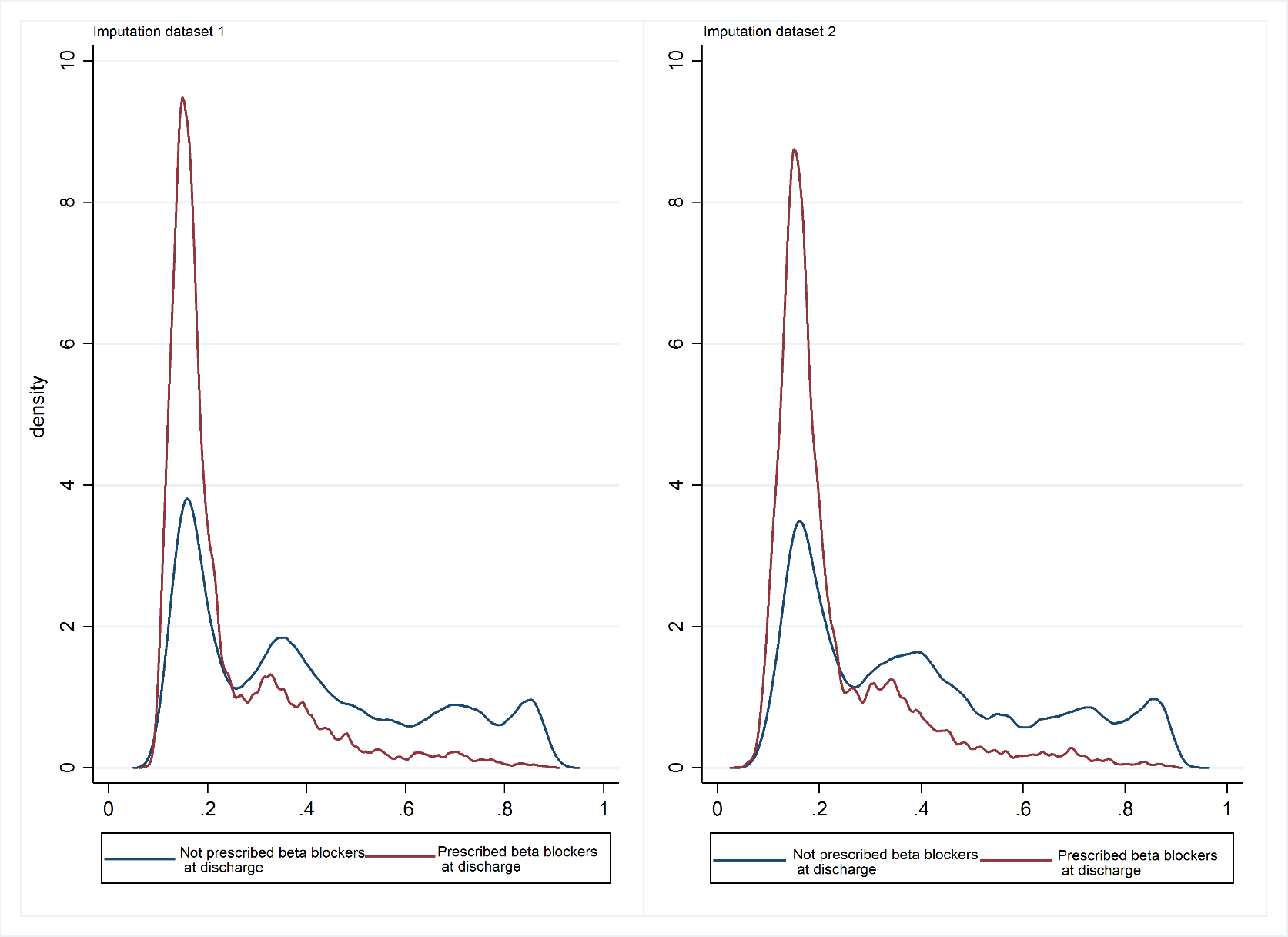
**

**Figure 2.** Overlap assumption assessment plots for imputation 3 and 4

**
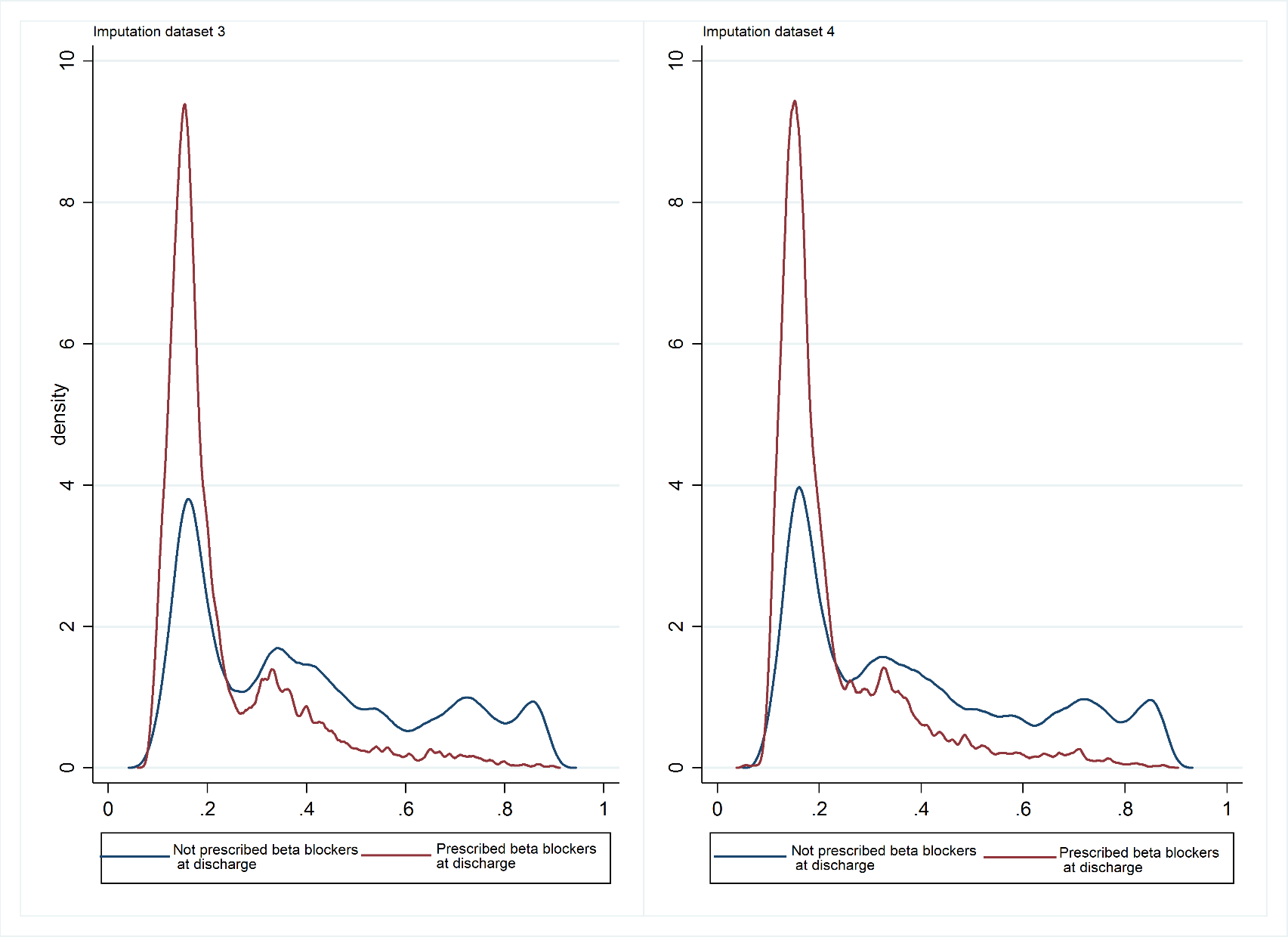
**

**Figure 3.** Overlap assumption assessment plots for imputation 5 and 6

**
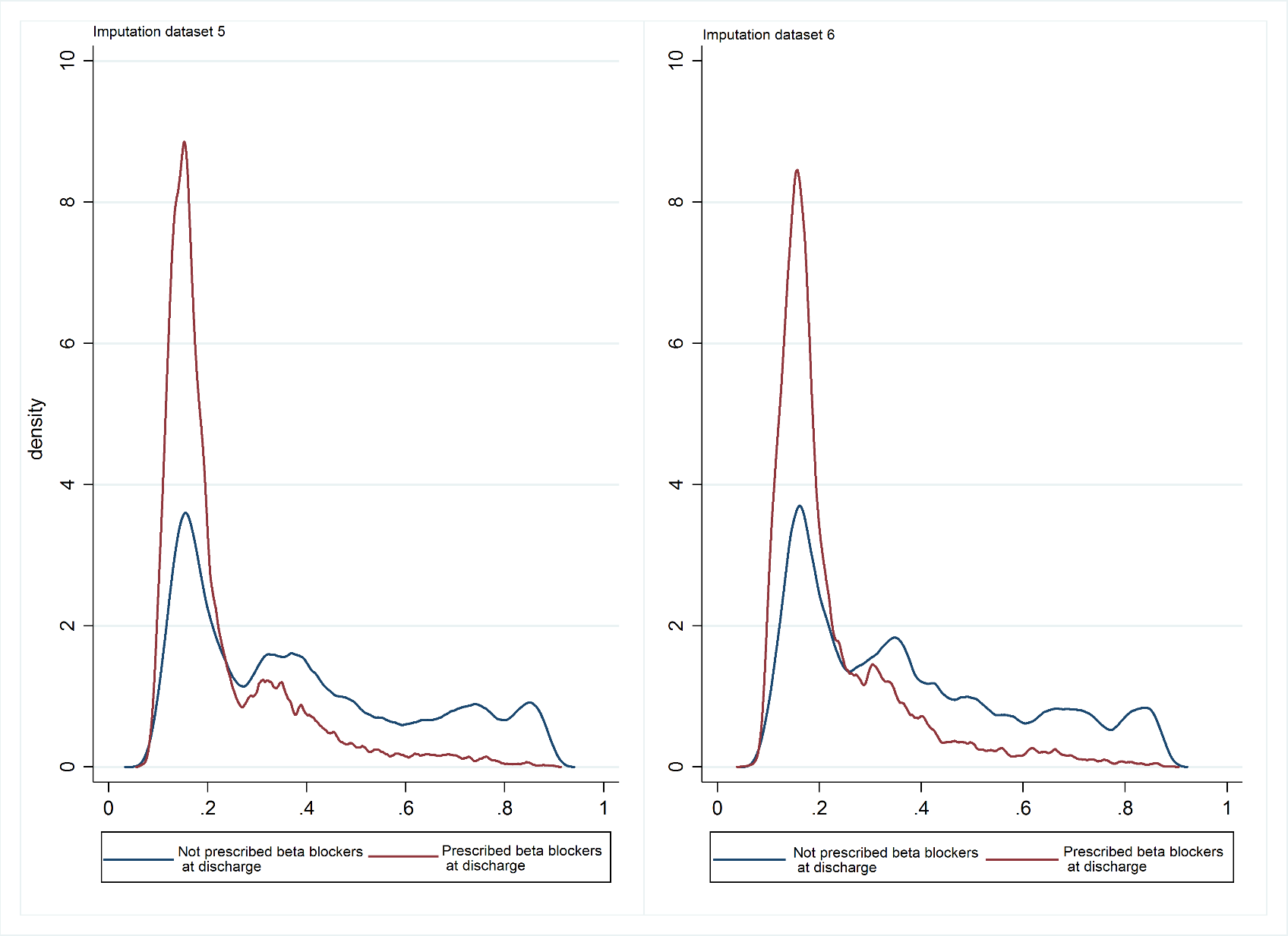
**

**Figure 4.** Overlap assumption assessment plots for imputation 7 and 8

**
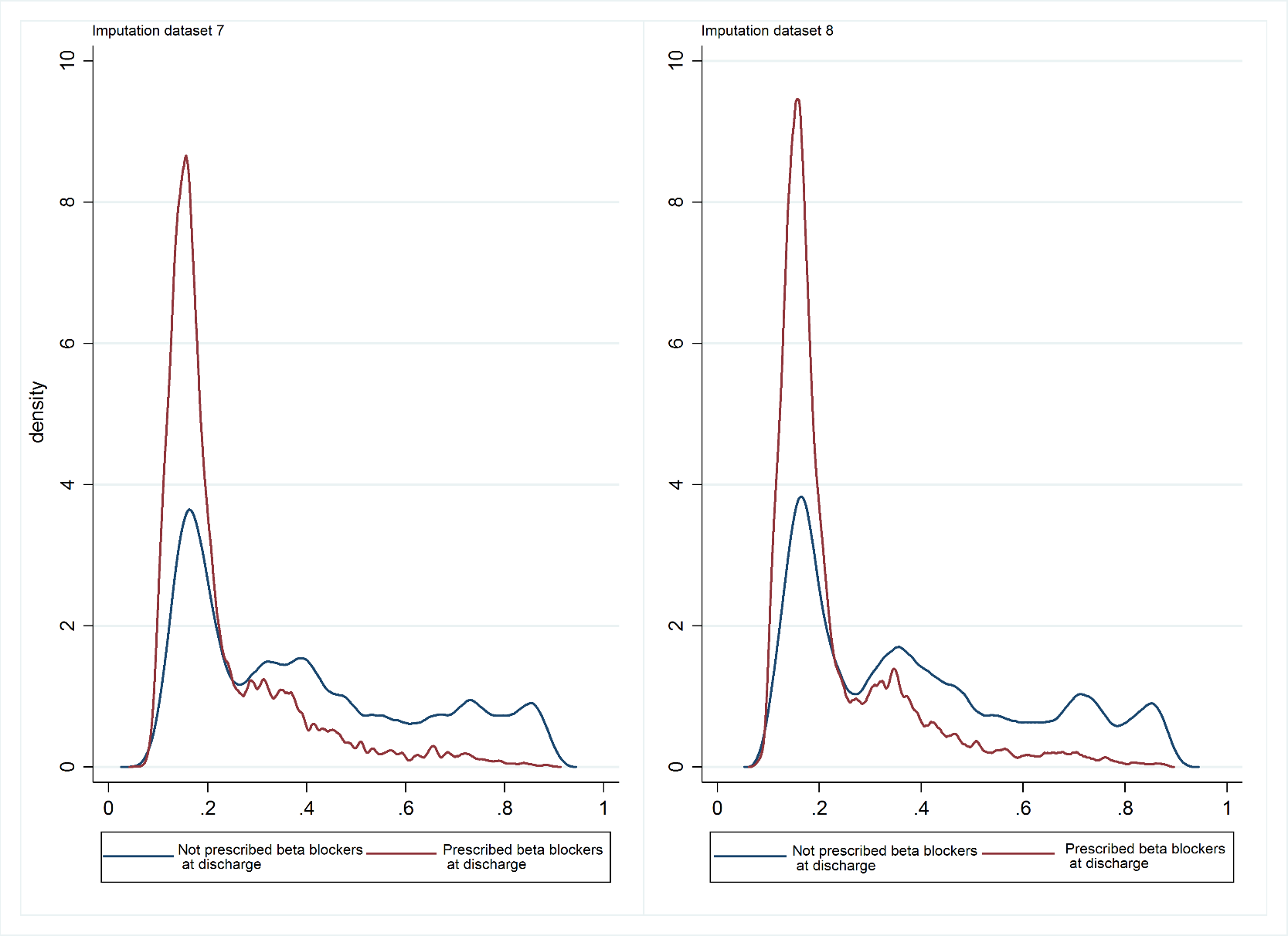
**

**Figure 5.** Overlap assumption assessment plots for imputation 9 and 10


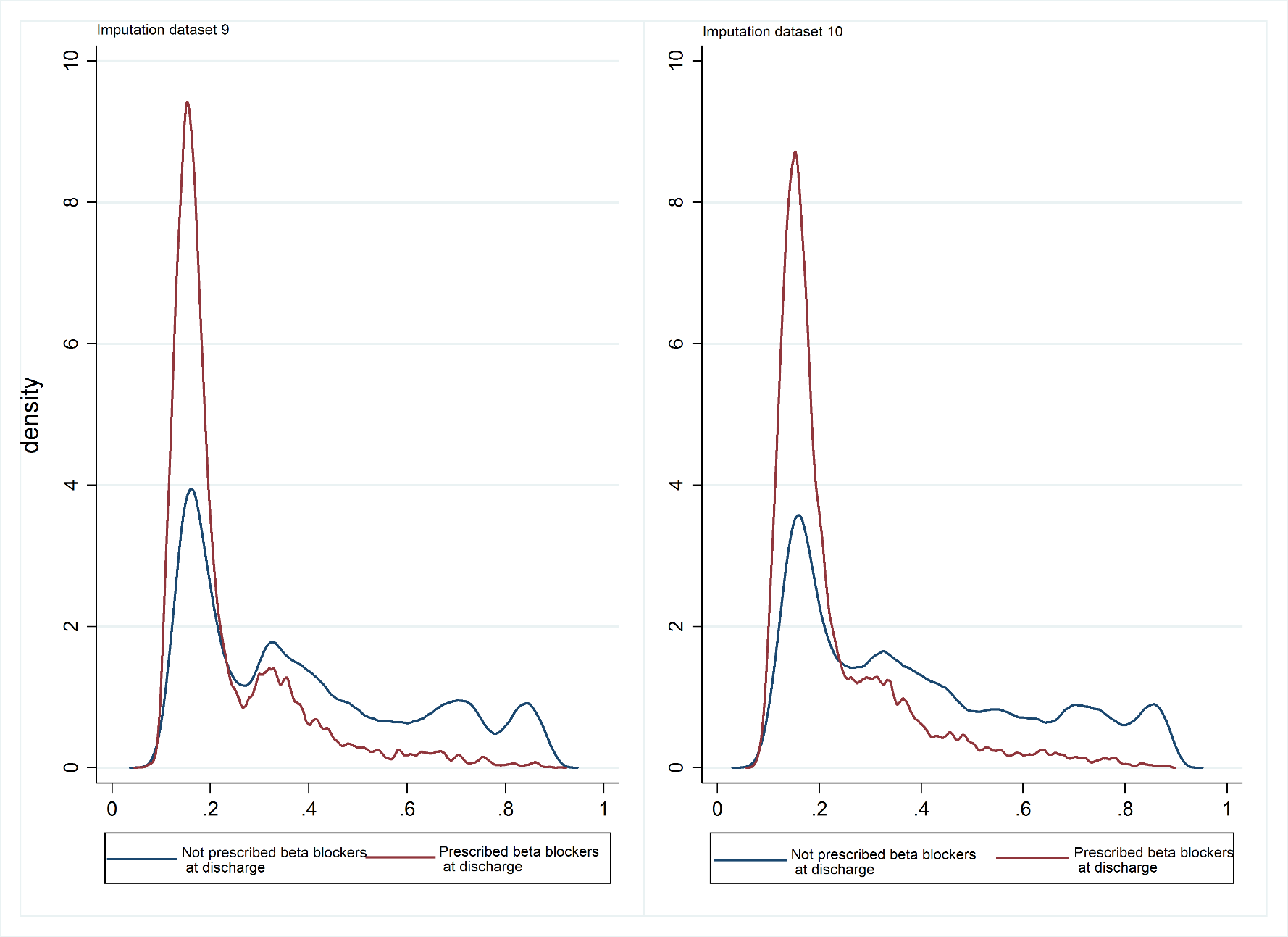


**Table 4.** Balance check parameters using standardized differences and variance ratios (imputation dataset 1)

|  | **Standardized differences** | | **Variance ratios** | |
| --- | --- | --- | --- | --- |
| **Data** | **Raw** | **Weighted** | **Raw** | **Weighted** |
| Age |  |  |  |  |
| Below 55 | ref | ref | ref | ref |
| 55-65 | 0.03 | 0.02 | 1.05 | 1.04 |
| 66-75 | 0.06 | 0.01 | 1.08 | 1.01 |
| 76-85 | 0.03 | -0.02 | 1.04 | 0.98 |
| Above 85 | -0.08 | -0.01 | 0.87 | 0.99 |
| Male | 0.02 | 0.001 | 0.99 | 1.00 |
| Deprivation (IMD) |  |  |  |  |
| Least deprived (1) | re | ref | re | ref |
| 2 | 0.02 | 0.02 | 1.03 | 1.03 |
| 3 | 0.03 | -0.002 | 1.05 | 1.00 |
| 4 | -0.02 | -0.01 | 0.97 | 0.99 |
| Most deprived (5) | -0.003 | -0.00001 | 0.99 | 1.00 |
| Year of admission |  |  |  |  |
| 2007 | ref | ref | ref | ref |
| 2008 | 0.05 | 0.01 | 1.08 | 1.01 |
| 2009 | -0.02 | 0.02 | 0.96 | 1.03 |
| 2010 | -0.02 | 0.01 | 0.96 | 1.02 |
| 2011 | -0.04 | -0.02 | 0.90 | 0.95 |
| 2012 | -0.05 | -0.001 | 0.87 | 1.00 |
| 2013 | -0.06 | -0.01 | 0.71 | 0.93 |
| **Cardiovascular history** |  |  |  |  |
| Cerebrovascular disease | 0.003 | 0.003 | 1.01 | 1.01 |
| Peripheral vascular disease | 0.05 | 0.01 | 1.31 | 1.05 |
| **Cardiovascular risk factors** |  |  |  |  |
| Diabetes | 0.05 | 0.02 | 1.10 | 1.03 |
| Hypercholesterolaemia | 0.05 | -0.01 | 1.07 | 0.99 |
| Hypertension | 0.07 | 0.003 | 1.03 | 1.00 |
| Current or ex-smoker | 0.07 | 1.56x10^-6^ | 0.98 | 1.00 |
| Asthma or COPD | 0.07 | 0.002 | 1.05 | 1.00 |
| Family history of CHD | 0.05 | -0.002 | 1.08 | 1.00 |
| **Presenting characteristics** |  |  |  |  |
| Heart rate >110 bpm | -0.03 | -0.03 | 0.90 | 0.93 |
| Creatinine >200 (μmol/l) | -0.03 | -0.02 | 0.86 | 0.91 |
| Peak troponin | 0.02 | 0.01 | 0.94 | 0.98 |
| Cardiac arrest | 0.01 | -0.02 | 1.11 | 0.86 |
| **Electrocardiographic characteristics** |  |  |  |  |
| ST-segment deviation | 0.03 | -0.01 | 1.01 | 1.00 |
| Care by cardiologist | -0.01 | -0.01 | 1.00 | 1.00 |
| **Medication at discharge** |  |  |  |  |
| Aspirin |  |  |  |  |
| Received | 0.46 | 0.03 | 0.44 | 0.94 |
| Contraindicated/ not applicable | 0.01 | 0.01 | 1.07 | 1.07 |
| P2Y_12_ inhibitors |  |  |  |  |
| Received | 0.58 | 0.02 | 0.63 | 0.98 |
| Contraindicated/ not applicable | 0.05 | 0.02 | 1.28 | 1.09 |
| ACEi/ARBs |  |  |  |  |
| Received | 0.45 | 0.01 | 1.25 | 1.00 |
| Contraindicated/ not applicable | 0.12 | 0.01 | 2.27 | 1.05 |
| Statins |  |  |  |  |
| Received | 0.61 | 0.03 | 0.50 | 0.96 |
| Contraindicated/ not applicable | 0.04 | -0.001 | 1.43 | 0.99 |
| **In-hospital procedures** |  |  |  |  |
| Coronary angiography |  |  |  |  |
| Received | 0.13 | -0.001 | 0.97 | 1.00 |
| Contraindicated/ not applicable | -0.03 | 0.003 | 0.88 | 1.01 |
| Coronary intervention (PCI/CABG) |  |  |  |  |
| Received | 0.06 | 0.002 | 1.03 | 1.00 |
| Contraindicated/ not applicable | 0.0002 | 0.01 | 1.00 | 1.02 |

**Abbreviations:** ACE, angiotensin converting enzyme; ARB, angiotensin receptor blocker; CABG, coronary artery bypass graft; CHD, coronary heart disease; COPD, chronic obstructive pulmonary disease; IMD, Index of multiple deprivation; PCI, percutaneous coronary intervention; ref, reference category.

**Table 5.** Balance check parameters using standardized differences and variance ratios (imputation dataset 2)

|  | **Standardized differences** | | **Variance ratios** | |
| --- | --- | --- | --- | --- |
| **Data** | **Raw** | **Weighted** | **Raw** | **Weighted** |
| Age |  |  |  |  |
| Below 55 | ref | ref | ref | ref |
| 55-65 | 0.02 | 0.01 | 1.04 | 1.02 |
| 66-75 | 0.07 | 0.003 | 1.10 | 1.00 |
| 76-85 | 0.03 | -0.004 | 1.03 | 1.00 |
| Above 85 | -0.08 | -0.02 | 0.87 | 0.97 |
| Male | 0.02 | -0.01 | 0.99 | 1.00 |
| Deprivation (IMD) |  |  |  |  |
| Least deprived (1) | ref | ref | ref | ref |
| 2 | 0.02 | 0.01 | 1.02 | 1.01 |
| 3 | 0.04 | -0.004 | 1.06 | 1.00 |
| 4 | 0.004 | -0.003 | 1.01 | 1.00 |
| Most deprived (5) | -0.02 | 0.01 | 0.98 | 1.01 |
| Year of admission |  |  |  |  |
| 2007 | ref | ref | ref | ref |
| 2008 | 0.05 | 0.004 | 1.07 | 1.01 |
| 2009 | -0.02 | 0.0003 | 0.97 | 1.00 |
| 2010 | -0.03 | 0.0001 | 0.93 | 1.00 |
| 2011 | -0.05 | -0.01 | 0.89 | 0.97 |
| 2012 | -0.06 | -0.01 | 0.85 | 0.98 |
| 2013 | -0.05 | -0.03 | 0.74 | 0.83 |
| **Cardiovascular history** |  |  |  |  |
| Cerebrovascular disease | 0.01 | -0.003 | 1.03 | 0.99 |
| Peripheral vascular disease | 0.04 | 0.01 | 1.21 | 1.05 |
| **Cardiovascular risk factors** |  |  |  |  |
| Diabetes | 0.03 | 0.02 | 1.05 | 1.03 |
| Hypercholesterolaemia | 0.06 | -0.002 | 1.08 | 1.00 |
| Hypertension | 0.07 | 0.001 | 1.03 | 1.00 |
| Current or ex-smoker | 0.09 | 0.02 | 0.97 | 0.99 |
| Asthma or COPD | 0.06 | -0.002 | 1.04 | 1.00 |
| Family history of CHD | 0.06 | -0.002 | 1.09 | 1.00 |
| **Presenting characteristics** |  |  |  |  |
| Heart rate >110 bpm | -0.06 | -0.003 | 0.84 | 0.99 |
| Creatinine >200 (μmol/l) | -0.04 | -0.002 | 0.77 | 0.99 |
| Peak troponin | 0.02 | 0.02 | 0.94 | 0.94 |
| Cardiac arrest | 0.03 | -0.01 | 1.19 | 0.95 |
| **Electrocardiographic characteristics** |  |  |  |  |
| ST-segment deviation | 0.03 | -0.006 | 1.01 | 1.00 |
| Care by cardiologist | -0.02 | -0.04 | 1.01 | 1.02 |
| **Medication at discharge** |  |  |  |  |
| Aspirin |  |  |  |  |
| Received | 0.46 | 0.03 | 0.43 | 0.94 |
| Contraindicated/ not applicable | 0.01 | -0.01 | 1.09 | 0.97 |
| P2Y_12_ inhibitors |  |  |  |  |
| Received | 0.56 | 0.02 | 0.63 | 0.98 |
| Contraindicated/ not applicable | 0.04 | 0.004 | 1.24 | 1.02 |
| ACEi/ARBs | 0.11 | -0.02 | 1.11 | 0.98 |
| Received | 0.44 | -0.01 | 1.24 | 1.00 |
| Contraindicated/ not applicable | 0.10 | 0.03 | 1.99 | 1.20 |
| Statins |  |  |  |  |
| Received | 0.61 | 0.02 | 0.50 | 0.97 |
| Contraindicated/ not applicable | 0.03 | 0.004 | 1.26 | 1.03 |
| **In-hospital procedures** |  |  |  |  |
| Coronary angiography |  |  |  |  |
| Received | 0.16 | -0.01 | 0.96 | 1.00 |
| Contraindicated/ not applicable | -0.04 | 0.002 | 0.84 | 1.01 |
| Coronary intervention (PCI/CABG) |  |  |  |  |
| Received | 0.09 | -0.01 | 1.06 | 1.00 |
| Contraindicated/ not applicable | 0.003 | 0.002 | 1.01 | 1.01 |

**Abbreviations:** ACE, angiotensin converting enzyme; ARB, angiotensin receptor blocker; CABG, coronary artery bypass graft; CHD, coronary heart disease; COPD, chronic obstructive pulmonary disease; IMD, Index of multiple deprivation; PCI, percutaneous coronary intervention; ref, reference category.

**Table 6.** Balance check parameters using standardized differences and variance ratios (imputation dataset 3)

|  | **Standardized differences** | | **Variance ratios** | |
| --- | --- | --- | --- | --- |
| **Data** | **Raw** | **Weighted** | **Raw** | **Weighted** |
| Age |  |  |  |  |
| Below 55 | ref | ref | ref | ref |
| 55-65 | 0.03 | 0.01 | 1.05 | 1.02 |
| 66-75 | 0.06 | 0.002 | 1.09 | 1.00 |
| 76-85 | 0.03 | -0.01 | 1.03 | 0.99 |
| Above 85 | -0.08 | -0.02 | 0.88 | 0.96 |
| Male | 0.02 | 0.004 | 0.99 | 1.00 |
| Deprivation (IMD) |  |  |  |  |
| Least deprived (1) | ref | ref | ref | ref |
| 2 | -0.01 | 0.01 | 0.99 | 1.01 |
| 3 | 0.05 | 0.002 | 1.07 | 1.00 |
| 4 | -0.01 | -0.01 | 0.98 | 0.99 |
| Most deprived (5) | -0.01 | 0.01 | 0.98 | 1.01 |
| Year of admission |  |  |  |  |
| 2007 | ref | ref | ref | ref |
| 2008 | 0.05 | 0.001 | 1.08 | 1.00 |
| 2009 | -0.02 | 0.01 | 0.97 | 1.01 |
| 2010 | -0.01 | -0.02 | 0.97 | 0.95 |
| 2011 | -0.05 | -0.01 | 0.89 | 0.98 |
| 2012 | -0.07 | 0.001 | 0.83 | 1.00 |
| 2013 | -0.05 | -0.01 | 0.75 | 0.93 |
| **Cardiovascular history** |  |  |  |  |
| Cerebrovascular disease | -0.0001 | -0.003 | 0.99 | 0.99 |
| Peripheral vascular disease | 0.05 | 0.01 | 1.27 | 1.06 |
| **Cardiovascular risk factors** |  |  |  |  |
| Diabetes | 0.04 | 0.02 | 1.07 | 1.04 |
| Hypercholesterolaemia | 0.06 | -0.01 | 1.08 | 0.98 |
| Hypertension | 0.06 | -0.01 | 1.03 | 0.99 |
| Current or ex-smoker | 0.09 | 0.01 | 0.97 | 1.00 |
| Asthma or COPD | 0.08 | 0.002 | 1.06 | 1.00 |
| Family history of CHD | 0.06 | 0.001 | 1.09 | 1.00 |
| **Presenting characteristics** |  |  |  |  |
| Heart rate >110 bpm | -0.05 | -0.01 | 0.88 | 0.97 |
| Creatinine >200 (μmol/l) | -0.04 | -0.01 | 0.80 | 0.94 |
| Peak troponin | -0.003 | -0.001 | 1.01 | 1.00 |
| Cardiac arrest | 0.03 | 0.02 | 1.23 | 1.14 |
| **Electrocardiographic characteristics** |  |  |  |  |
| ST-segment deviation | 0.03 | -0.003 | 1.01 | 1.00 |
| Care by cardiologist | -0.01 | -0.02 | 1.01 | 1.02 |
| **Medication at discharge** |  |  |  |  |
| Aspirin |  |  |  |  |
| Received | 0.45 | 0.03 | 0.45 | 0.95 |
| Contraindicated/ not applicable | 0.03 | 0.003 | 1.17 | 1.01 |
| P2Y_12_ inhibitors |  |  |  |  |
| Received | 0.56 | 0.02 | 0.64 | 0.98 |
| Contraindicated/ not applicable | 0.06 | 0.001 | 1.37 | 1.01 |
| ACEi/ARBs |  |  |  |  |
| Received | 0.44 | 0.01 | 1.25 | 1.00 |
| Contraindicated/ not applicable | 0.12 | 0.01 | 2.31 | 1.04 |
| Statins |  |  |  |  |
| Received | 0.62 | 0.03 | 0.50 | 0.96 |
| Contraindicated/ not applicable | 0.05 | 0.002 | 1.63 | 1.02 |
| **In-hospital procedures** |  |  |  |  |
| Coronary angiography |  |  |  |  |
| Received | 0.13 | 0.003 | 0.97 | 1.00 |
| Contraindicated/ not applicable | -0.04 | -0.003 | 0.85 | 0.99 |
| Coronary intervention (PCI/CABG) |  |  |  |  |
| Received | 0.06 | -0.003 | 1.04 | 1.00 |
| Contraindicated/ not applicable | -0.01 | 0.01 | 0.96 | 1.02 |

**Abbreviations:** ACE, angiotensin converting enzyme; ARB, angiotensin receptor blocker; CABG, coronary artery bypass graft; CHD, coronary heart disease; COPD, chronic obstructive pulmonary disease; IMD, Index of multiple deprivation; PCI, percutaneous coronary intervention; ref, reference category.

**Table 7.** Balance check parameters using standardized differences and variance ratios (imputation dataset 4)

|  | **Standardized differences** | | **Variance ratios** | |
| --- | --- | --- | --- | --- |
| **Data** | **Raw** | **Weighted** | **Raw** | **Weighted** |
| Age |  |  |  |  |
| Below 55 | ref | ref | ref | ref |
| 55-65 | 0.01 | 0.02 | 1.02 | 1.04 |
| 66-75 | 0.06 | -0.02 | 1.09 | 0.97 |
| 76-85 | 0.03 | -0.01 | 1.03 | 0.99 |
| Above 85 | -0.08 | -0.01 | 0.87 | 0.99 |
| Male | 0.01 | -0.01 | 1.00 | 1.00 |
| Deprivation (IMD) |  |  |  |  |
| Least deprived (1) | ref | ref | ref | ref |
| 2 | 0.02 | -0.01 | 1.03 | 0.99 |
| 3 | 0.03 | 0.02 | 1.04 | 1.03 |
| 4 | -0.01 | 0.004 | 0.99 | 1.01 |
| Most deprived (5) | -0.01 | 0.01 | 0.98 | 1.02 |
| Year of admission |  |  |  |  |
| 2007 | ref | ref | ref | ref |
| 2008 | 0.05 | 0.01 | 1.08 | 1.01 |
| 2009 | -0.01 | -0.02 | 0.98 | 0.97 |
| 2010 | -0.02 | 0.01 | 0.95 | 1.02 |
| 2011 | -0.07 | 0.01 | 0.86 | 1.01 |
| 2012 | -0.06 | -0.01 | 0.84 | 0.99 |
| 2013 | -0.06 | -0.01 | 0.72 | 0.95 |
| **Cardiovascular history** |  |  |  |  |
| Cerebrovascular disease | -0.003 | -0.002 | 0.99 | 0.99 |
| Peripheral vascular disease | 0.05 | 0.003 | 1.28 | 1.02 |
| **Cardiovascular risk factors** |  |  |  |  |
| Diabetes | 0.03 | 0.01 | 1.06 | 1.01 |
| Hypercholesterolaemia | 0.06 | -0.01 | 1.08 | 0.98 |
| Hypertension | 0.06 | 0.004 | 1.03 | 1.00 |
| Current or ex-smoker | 0.09 | 0.01 | 0.97 | 1.00 |
| Asthma or COPD | 0.06 | 0.01 | 1.04 | 1.00 |
| Family history of CHD | 0.07 | 0.01 | 1.11 | 1.02 |
| **Presenting characteristics** |  |  |  |  |
| Heart rate >110 bpm | -0.07 | -0.01 | 1.01 | 1.00 |
| Creatinine >200 (μmol/l) | -0.05 | 0.004 | 0.94 | 1.02 |
| Peak troponin | 0.02 | -0.02 | 0.94 | 1.04 |
| Cardiac arrest | 0.02 | -0.02 | 1.16 | 0.87 |
| **Electrocardiographic characteristics** |  |  |  |  |
| ST-segment deviation | 0.02 | -0.003 | 1.01 | 1.00 |
| Care by cardiologist | -0.01 | -0.01 | 1.00 | 1.01 |
| **Medication at discharge** |  |  |  |  |
| Aspirin |  |  |  |  |
| Received | 0.47 | 0.03 | 0.43 | 0.94 |
| Contraindicated/ not applicable | 0.01 | -0.004 | 1.04 | 0.98 |
| P2Y_12_ inhibitors |  |  |  |  |
| Received | 0.54 | 0.03 | 0.64 | 0.96 |
| Contraindicated/ not applicable | 0.07 | -0.02 | 1.42 | 0.98 |
| ACEi/ARBs |  |  |  |  |
| Received | 0.45 | 0.02 | 1.27 | 1.01 |
| Contraindicated/ not applicable | 0.12 | -0.02 | 2.24 | 0.88 |
| Statins |  |  |  |  |
| Received | 0.63 | 0.04 | 0.50 | 0.95 |
| Contraindicated/ not applicable | 0.03 | -0.03 | 1.42 | 0.77 |
| **In-hospital procedures** |  |  |  |  |
| Coronary angiography |  |  |  |  |
| Received | 0.14 | -0.002 | 0.97 | 1.00 |
| Contraindicated/ not applicable | -0.04 | -0.001 | 0.86 | 1.00 |
| Coronary intervention (PCI/CABG) |  |  |  |  |
| Received | 0.07 | 0.002 | 1.04 | 1.00 |
| Contraindicated/ not applicable | -0.01 | 0.01 | 0.97 | 1.03 |

**Abbreviations:** ACE, angiotensin converting enzyme; ARB, angiotensin receptor blocker; CABG, coronary artery bypass graft; CHD, coronary heart disease; COPD, chronic obstructive pulmonary disease; IMD, Index of multiple deprivation; PCI, percutaneous coronary intervention; ref, reference category.

**Table 8.** Balance check parameters using standardized differences and variance ratios (imputation dataset 5)

|  | **Standardized differences** | | **Variance ratios** | |
| --- | --- | --- | --- | --- |
| **Data** | **Raw** | **Weighted** | **Raw** | **Weighted** |
| Age |  |  |  |  |
| Below 55 | ref | ref | ref | ref |
| 55-65 | 0.03 | 0.01 | 1.05 | 1.02 |
| 66-75 | 0.07 | -0.001 | 1.10 | 1.00 |
| 76-85 | 0.02 | -0.02 | 1.02 | 0.98 |
| Above 85 | -0.09 | 0.001 | 0.87 | 1.00 |
| Male | 0.01 | 0.0001 | 1.00 | 1.00 |
| Deprivation (IMD) |  |  |  |  |
| Least deprived (1) | ref | ref | ref | ref |
| 2 | 0.03 | -0.002 | 1.04 | 1.00 |
| 3 | 0.04 | -0.01 | 1.05 | 1.00 |
| 4 | -0.03 | 0.01 | 0.96 | 1.01 |
| Most deprived (5) | -0.01 | 0.01 | 0.98 | 1.01 |
| Year of admission |  |  |  |  |
| 2007 | ref | ref | ref | ref |
| 2008 | 0.06 | 0.004 | 1.09 | 1.01 |
| 2009 | -0.02 | 0.01 | 0.97 | 1.01 |
| 2010 | -0.04 | 0.004 | 0.92 | 1.01 |
| 2011 | -0.05 | -0.01 | 0.89 | 0.97 |
| 2012 | -0.06 | -0.01 | 0.83 | 0.96 |
| 2013 | -0.05 | -0.02 | 0.77 | 0.91 |
| **Cardiovascular history** |  |  |  |  |
| Cerebrovascular disease | 0.01 | -0.02 | 1.01 | 0.95 |
| Peripheral vascular disease | 0.05 | 0.01 | 1.28 | 1.06 |
| **Cardiovascular risk factors** |  |  |  |  |
| Diabetes | 0.03 | 0.03 | 1.06 | 1.05 |
| Hypercholesterolaemia | 0.05 | -0.01 | 1.06 | 0.99 |
| Hypertension | 0.05 | 0.002 | 1.02 | 1.00 |
| Current or ex-smoker | 0.08 | 0.0002 | 0.98 | 1.00 |
| Asthma or COPD | 0.08 | 0.0003 | 1.06 | 1.00 |
| Family history of CHD | 0.05 | 0.002 | 1.07 | 1.00 |
| **Presenting characteristics** |  |  |  |  |
| Heart rate >110 bpm | -0.05 | -0.01 | 0.88 | 0.98 |
| Creatinine >200 (μmol/l) | -0.04 | -0.003 | 0.79 | 0.99 |
| Peak troponin | 0.01 | -0.002 | 0.97 | 1.00 |
| Cardiac arrest | 0.03 | -0.03 | 1.23 | 0.84 |
| **Electrocardiographic characteristics** |  |  |  |  |
| ST-segment deviation | 0.04 | -0.001 | 1.01 | 1.00 |
| Care by cardiologist | -0.01 | -0.03 | 1.00 | 1.02 |
| **Medication at discharge** |  |  |  |  |
| Aspirin |  |  |  |  |
| Received | 0.46 | 0.02 | 0.44 | 0.97 |
| Contraindicated/ not applicable | 0.02 | 0.01 | 1.13 | 1.06 |
| P2Y_12_ inhibitors |  |  |  |  |
| Received | 0.58 | 0.02 | 0.63 | 0.98 |
| Contraindicated/ not applicable | 0.07 | 0.01 | 1.41 | 1.04 |
| ACEi/ARBs |  |  |  |  |
| Received | 0.44 | 0.01 | 1.26 | 1.00 |
| Contraindicated/ not applicable | 0.10 | 0.01 | 2.03 | 1.09 |
| Statins |  |  |  |  |
| Received | 0.63 | 0.02 | 0.50 | 0.97 |
| Contraindicated/ not applicable | 0.02 | -0.01 | 1.20 | 0.95 |
| **In-hospital procedures** |  |  |  |  |
| Coronary angiography |  |  |  |  |
| Received | 0.16 | -0.01 | 0.96 | 1.00 |
| Contraindicated/ not applicable | -0.04 | -0.01 | 0.83 | 0.98 |
| Coronary intervention (PCI/CABG) |  |  |  |  |
| Received | 0.07 | 0.01 | 1.04 | 1.00 |
| Contraindicated/ not applicable | -0.01 | -0.01 | 0.97 | 0.98 |

**Abbreviations:** ACE, angiotensin converting enzyme; ARB, angiotensin receptor blocker; CABG, coronary artery bypass graft; CHD, coronary heart disease; COPD, chronic obstructive pulmonary disease; IMD, Index of multiple deprivation; PCI, percutaneous coronary intervention; ref, reference category.

**Table 9.** Balance check parameters using standardized differences and variance ratios (imputation dataset 6)

|  | **Standardized differences** | | **Variance ratios** | |
| --- | --- | --- | --- | --- |
| **Data** | **Raw** | **Weighted** | **Raw** | **Weighted** |
| Age |  |  |  |  |
| Below 55 | ref | ref | ref | ref |
| 55-65 | 0.04 | 0.02 | 1.08 | 1.04 |
| 66-75 | 0.06 | -0.01 | 1.08 | 0.99 |
| 76-85 | 0.03 | -0.02 | 1.03 | 0.98 |
| Above 85 | -0.09 | 0.001 | 0.86 | 1.00 |
| Male | 0.05 | -0.01 | 0.99 | 1.00 |
| Deprivation (IMD) |  |  |  |  |
| Least deprived (1) | ref | ref | ref | ref |
| 2 | 0.03 | -0.001 | 1.04 | 1.00 |
| 3 | 0.04 | 0.01 | 1.06 | 1.01 |
| 4 | -0.03 | 0.01 | 0.95 | 1.01 |
| Most deprived (5) | -0.01 | -0.02 | 0.98 | 0.97 |
| Year of admission |  |  |  |  |
| 2007 | ref | ref | ref | ref |
| 2008 | 0.06 | 0.004 | 1.10 | 1.01 |
| 2009 | -0.02 | 0.02 | 0.97 | 1.04 |
| 2010 | -0.03 | -0.03 | 0.94 | 0.94 |
| 2011 | -0.05 | -0.01 | 0.88 | 0.98 |
| 2012 | -0.06 | -0.002 | 0.84 | 0.99 |
| 2013 | -0.06 | -0.01 | 0.69 | 0.92 |
| **Cardiovascular history** |  |  |  |  |
| Cerebrovascular disease | -0.01 | -0.01 | 0.98 | 0.97 |
| Peripheral vascular disease | 0.05 | 0.002 | 1.33 | 1.01 |
| **Cardiovascular risk factors** |  |  |  |  |
| Diabetes | 0.03 | 0.02 | 1.06 | 1.03 |
| Hypercholesterolaemia | 0.05 | -0.01 | 1.07 | 0.99 |
| Hypertension | 0.07 | 0.01 | 1.03 | 1.00 |
| Current or ex-smoker | 0.08 | 0.01 | 0.97 | 1.00 |
| Asthma or COPD | 0.06 | -0.004 | 1.04 | 1.00 |
| Family history of CHD | 0.06 | 0.01 | 1.09 | 1.01 |
| **Presenting characteristics** |  |  |  |  |
| Heart rate >110 bpm | -0.05 | -0.04 | 0.85 | 0.88 |
| Creatinine >200 (μmol/l) | -0.05 | -0.001 | 0.75 | 0.99 |
| Peak troponin | 0.01 | 0.003 | 0.97 | 0.99 |
| Cardiac arrest | 0.02 | -0.02 | 1.19 | 0.89 |
| **Electrocardiographic characteristics** |  |  |  |  |
| ST-segment deviation | 0.05 | -0.01 | 1.02 | 1.00 |
| Care by cardiologist | -0.02 | -0.01 | 1.02 | 1.01 |
| **Medication at discharge** |  |  |  |  |
| Aspirin |  |  |  |  |
| Received | 0.46 | 0.04 | 0.44 | 0.93 |
| Contraindicated/ not applicable | 0.03 | 0.001 | 1.17 | 1.00 |
| P2Y_12_ inhibitors |  |  |  |  |
| Received | 0.53 | 0.03 | 0.66 | 0.97 |
| Contraindicated/ not applicable | 0.08 | 0.01 | 1.52 | 1.05 |
| ACEi/ARBs |  |  |  |  |
| Received | 0.43 | 0.02 | 1.24 | 1.01 |
| Contraindicated/ not applicable | 0.10 | -0.03 | 1.98 | 0.85 |
| Statins |  |  |  |  |
| Received | 0.61 | 0.05 | 0.51 | 0.94 |
| Contraindicated/ not applicable | 0.06 | -0.06 | 1.62 | 0.65 |
| **In-hospital procedures** |  |  |  |  |
| Coronary angiography |  |  |  |  |
| Received | 0.15 | -0.01 | 0.96 | 1.00 |
| Contraindicated/ not applicable | -0.03 | -0.004 | 0.88 | 0.98 |
| Coronary intervention (PCI/CABG) |  |  |  |  |
| Received | 0.09 | -0.01 | 1.06 | 0.99 |
| Contraindicated/ not applicable | -0.02 | 0.0003 | 0.94 | 1.00 |

**Abbreviations:** ACE, angiotensin converting enzyme; ARB, angiotensin receptor blocker; CABG, coronary artery bypass graft; CHD, coronary heart disease; COPD, chronic obstructive pulmonary disease; IMD, Index of multiple deprivation; PCI, percutaneous coronary intervention; ref, reference category.

**Table 10.** Balance check parameters using standardized differences and variance ratios (imputation dataset 7)

|  | **Standardized differences** | | **Variance ratios** | |
| --- | --- | --- | --- | --- |
| **Data** | **Raw** | **Weighted** | **Raw** | **Weighted** |
| Age |  |  |  |  |
| Below 55 | ref | ref | ref | ref |
| 55-65 | 0.02 | 0.02 | 1.04 | 1.03 |
| 66-75 | 0.06 | 0.02 | 1.09 | 1.03 |
| 76-85 | 0.02 | -0.03 | 1.03 | 0.97 |
| Above 85 | -0.08 | 0.01 | 0.88 | 1.01 |
| Male | 0.03 | -0.001 | 0.99 | 1.00 |
| Deprivation (IMD) |  |  |  |  |
| Least deprived (1) | ref | ref | ref | ref |
| 2 | 0.01 | 0.01 | 1.02 | 1.02 |
| 3 | 0.03 | -0.002 | 1.04 | 1.00 |
| 4 | -0.01 | 0.01 | 0.99 | 1.02 |
| Most deprived (5) | -0.01 | 0.003 | 0.99 | 1.00 |
| Year of admission |  |  |  |  |
| 2007 | ref | ref | ref | ref |
| 2008 | 0.04 | 0.02 | 1.06 | 1.03 |
| 2009 | -0.02 | 0.02 | 0.97 | 1.03 |
| 2010 | -0.01 | -0.03 | 0.98 | 0.93 |
| 2011 | -0.05 | -0.01 | 0.89 | 0.98 |
| 2012 | -0.05 | -0.01 | 0.86 | 0.98 |
| 2013 | -0.04 | -0.02 | 0.77 | 0.89 |
| **Cardiovascular history** |  |  |  |  |
| Cerebrovascular disease | -0.004 | -0.02 | 0.99 | 0.95 |
| Peripheral vascular disease | 0.06 | 0.01 | 1.34 | 1.06 |
| **Cardiovascular risk factors** |  |  |  |  |
| Diabetes | 0.04 | 0.02 | 1.08 | 1.03 |
| Hypercholesterolaemia | 0.06 | 0.004 | 1.08 | 1.01 |
| Hypertension | 0.06 | 0.001 | 1.03 | 1.00 |
| Current or ex-smoker | 0.07 | 0.02 | 0.97 | 0.99 |
| Asthma or COPD | 0.07 | -0.01 | 1.05 | 1.00 |
| Family history of CHD | 0.05 | -0.02 | 1.08 | 0.97 |
| **Presenting characteristics** |  |  |  |  |
| Heart rate >110 bpm | -0.05 | -0.02 | 0.88 | 0.96 |
| Creatinine >200 (μmol/l) | -0.04 | 0.002 | 0.82 | 1.01 |
| Peak troponin | -0.01 | -0.01 | 1.01 | 1.04 |
| Cardiac arrest | 0.03 | -0.01 | 1.20 | 0.91 |
| **Electrocardiographic characteristics** |  |  |  |  |
| ST-segment deviation | 0.04 | -0.003 | 1.01 | 1.00 |
| Care by cardiologist | 0.01 | -0.02 | 1.00 | 1.01 |
| **Medication at discharge** |  |  |  |  |
| Aspirin |  |  |  |  |
| Received | 0.47 | 0.02 | 0.43 | 0.96 |
| Contraindicated/ not applicable | 0.004 | 0.01 | 1.02 | 1.06 |
| P2Y_12_ inhibitors |  |  |  |  |
| Received | 0.55 | 0.02 | 0.65 | 0.98 |
| Contraindicated/ not applicable | 0.07 | -0.001 | 1.43 | 1.00 |
| ACEi/ARBs |  |  |  |  |
| Received | 0.45 | 0.01 | 1.27 | 1.00 |
| Contraindicated/ not applicable | 0.12 | -0.03 | 2.31 | 0.85 |
| Statins |  |  |  |  |
| Received | 0.61 | 0.03 | 0.51 | 0.96 |
| Contraindicated/ not applicable | 0.06 | -0.04 | 1.71 | 0.72 |
| **In-hospital procedures** |  |  |  |  |
| Coronary angiography |  |  |  |  |
| Received | 0.13 | -0.01 | 0.97 | 1.00 |
| Contraindicated/ not applicable | -0.03 | -0.01 | 0.86 | 0.98 |
| Coronary intervention (PCI/CABG) |  |  |  |  |
| Received | 0.07 | -0.003 | 1.04 | 1.00 |
| Contraindicated/ not applicable | 0.01 | 0.0002 | 1.02 | 1.00 |

**Abbreviations:** ACE, angiotensin converting enzyme; ARB, angiotensin receptor blocker; CABG, coronary artery bypass graft; CHD, coronary heart disease; COPD, chronic obstructive pulmonary disease; IMD, Index of multiple deprivation; PCI, percutaneous coronary intervention; ref, reference category.

**Table 11.** Balance check parameters using standardized differences and variance ratios (imputation dataset 8)

|  | **Standardized differences** | | **Variance ratios** | |
| --- | --- | --- | --- | --- |
| **Data** | **Raw** | **Weighted** | **Raw** | **Weighted** |
| Age |  |  |  |  |
| Below 55 | ref | ref | ref | ref |
| 55-65 | 0.02 | 0.02 | 1.04 | 1.03 |
| 66-75 | 0.06 | -0.01 | 1.08 | 0.99 |
| 76-85 | 0.02 | -0.01 | 1.03 | 0.99 |
| Above 85 | -0.07 | -0.01 | 0.89 | 0.98 |
| Male | 0.03 | 0.01 | 0.99 | 1.00 |
| Deprivation (IMD) |  |  |  |  |
| Least deprived (1) | ref | ref | ref | ref |
| 2 | 0.01 | 0.004 | 1.02 | 1.01 |
| 3 | 0.04 | -0.01 | 1.06 | 0.99 |
| 4 | -0.002 | 0.01 | 1.00 | 1.02 |
| Most deprived (5) | -0.02 | 0.001 | 0.97 | 1.00 |
| Year of admission |  |  |  |  |
| 2007 | ref | ref | ref | ref |
| 2008 | 0.05 | 0.01 | 1.08 | 1.02 |
| 2009 | -0.02 | 0.01 | 0.97 | 1.02 |
| 2010 | -0.03 | -0.003 | 0.94 | 0.99 |
| 2011 | -0.04 | -0.02 | 0.91 | 0.95 |
| 2012 | -0.06 | -0.008 | 0.84 | 0.98 |
| 2013 | -0.06 | -0.02 | 0.73 | 0.89 |
| **Cardiovascular history** |  |  |  |  |
| Cerebrovascular disease | 0.01 | 0.001 | 1.04 | 1.00 |
| Peripheral vascular disease | 0.05 | 0.003 | 1.27 | 1.02 |
| **Cardiovascular risk factors** |  |  |  |  |
| Diabetes | 0.03 | 0.01 | 1.05 | 1.03 |
| Hypercholesterolaemia | 0.04 | -0.003 | 1.06 | 1.00 |
| Hypertension | 0.07 | 0.004 | 1.03 | 1.00 |
| Current or ex-smoker | 0.06 | 0.01 | 0.98 | 0.99 |
| Asthma or COPD | 0.07 | 0.01 | 1.05 | 1.00 |
| Family history of CHD |  |  |  |  |
| **Presenting characteristics** |  |  |  |  |
| Heart rate >110 bpm | -0.06 | -0.01 | 0.83 | 0.97 |
| Creatinine >200 (μmol/l) | -0.04 | -0.0003 | 0.80 | 1.00 |
| Peak troponin | 0.0001 | -0.01 | 1.00 | 1.01 |
| Cardiac arrest | 0.02 | -0.01 | 1.17 | 0.90 |
| **Electrocardiographic characteristics** |  |  |  |  |
| ST-segment deviation | 0.03 | 0.01 | 1.01 | 1.00 |
| Care by cardiologist | -0.003 | -0.02 | 1.00 | 1.01 |
| **Medication at discharge** |  |  |  |  |
| Aspirin |  |  |  |  |
| Received | 0.45 | 0.03 | 0.44 | 0.94 |
| Contraindicated/ not applicable | 0.03 | 0.01 | 1.17 | 1.06 |
| P2Y_12_ inhibitors |  |  |  |  |
| Received | 0.56 | 0.02 | 0.65 | 0.98 |
| Contraindicated/ not applicable | 0.07 | 0.01 | 1.37 | 1.06 |
| ACEi/ARBs |  |  |  |  |
| Received | 045 | 0.01 | 1.26 | 1.00 |
| Contraindicated/ not applicable | 0.12 | -0.01 | 2.30 | 0.95 |
| Statins |  |  |  |  |
| Received | 0.62 | 0.03 | 0.50 | 0.96 |
| Contraindicated/ not applicable | 0.04 | -0.01 | 1.39 | 0.95 |
| **In-hospital procedures** |  |  |  |  |
| Coronary angiography |  |  |  |  |
| Received | 0.14 | -0.01 | 0.97 | 1.00 |
| Contraindicated/ not applicable | -0.03 | -0.01 | 0.88 | 0.97 |
| Coronary intervention (PCI/CABG) |  |  |  |  |
| Received | 0.06 | -0.01 | 1.03 | 0.99 |
| Contraindicated/ not applicable | -0.0003 | 0.0003 | 1.00 | 1.00 |

**Abbreviations:** ACE, angiotensin converting enzyme; ARB, angiotensin receptor blocker; CABG, coronary artery bypass graft; CHD, coronary heart disease; COPD, chronic obstructive pulmonary disease; IMD, Index of multiple deprivation; PCI, percutaneous coronary intervention; ref, reference category.

**Table 12.** Balance check parameters using standardized differences and variance ratios (imputation dataset 9)

|  | **Standardized differences** | | **Variance ratios** | |
| --- | --- | --- | --- | --- |
| **Data** | **Raw** | **Weighted** | **Raw** | **Weighted** |
| Age |  |  |  |  |
| Below 55 | ref | ref | ref | ref |
| 55-65 | 0.028 | 0.026 | 1.05 | 1.05 |
| 66-75 | 0.08 | 0.01 | 1.11 | 1.01 |
| 76-85 | 0.02 | -0.02 | 1.02 | 0.98 |
| Above 85 | -0.09 | -0.02 | 0.86 | 0.97 |
| Male | 0.02 | 0.001 | 0.99 | 1.00 |
| Deprivation (IMD) |  |  |  |  |
| Least deprived (1) | ref | ref | ref | ref |
| 2 | 0.01 | 0.004 | 1.01 | 1.01 |
| 3 | 0.04 | -0.02 | 1.06 | 0.97 |
| 4 | -0.02 | 0.01 | 0.97 | 1.01 |
| Most deprived (5) | -0.02 | -0.001 | 0.96 | 1.00 |
| Year of admission |  |  |  |  |
| 2007 | ref | ref | ref | ref |
| 2008 | 0.07 | -0.01 | 1.12 | 0.99 |
| 2009 | -0.02 | 0.01 | 0.97 | 1.01 |
| 2010 | -0.02 | 0.001 | 0.95 | 1.00 |
| 2011 | -0.05 | -0.01 | 0.89 | 0.97 |
| 2012 | -0.06 | -0.01 | 0.84 | 0.97 |
| 2013 | -0.06 | -0.02 | 0.72 | 0.92 |
| **Cardiovascular history** |  |  |  |  |
| Cerebrovascular disease | 0.01 | -0.003 | 1.02 | 0.99 |
| Peripheral vascular disease | 0.04 | 0.004 | 1.22 | 1.02 |
| **Cardiovascular risk factors** |  |  |  |  |
| Diabetes | 0.02 | 0.02 | 1.05 | 1.03 |
| Hypercholesterolaemia | 0.05 | -0.002 | 1.06 | 1.00 |
| Hypertension | 0.05 | 0.001 | 1.02 | 1.00 |
| Current or ex-smoker | 0.09 | 0.02 | 0.97 | 0.99 |
| Asthma or COPD | 0.06 | -0.01 | 1.04 | 0.99 |
| Family history of CHD | 0.06 | 0.001 | 1.09 | 1.00 |
| **Presenting characteristics** |  |  |  |  |
| Heart rate >110 bpm | -0.07 | -0.02 | 0.82 | 0.95 |
| Creatinine >200 (μmol/l) | -0.05 | -0.01 | 0.76 | 0.94 |
| Peak troponin | 0.01 | 0.01 | 0.97 | 0.98 |
| Cardiac arrest | 0.03 | -0.03 | 1.21 | 0.81 |
| **Electrocardiographic characteristics** |  |  |  |  |
| ST-segment deviation | 0.03 | 0.001 | 1.01 | 1.00 |
| Care by cardiologist | -0.02 | -0.02 | 1.02 | 1.01 |
| **Medication at discharge** |  |  |  |  |
| Aspirin |  |  |  |  |
| Received | 0.45 | 0.03 | 0.44 | 0.93 |
| Contraindicated/ not applicable | 0.03 | -0.001 | 1.18 | 0.99 |
| P2Y_12_ inhibitors |  |  |  |  |
| Received | 0.55 | 0.03 | 0.65 | 0.97 |
| Contraindicated/ not applicable | 0.08 | 0.01 | 1.47 | 1.04 |
| ACEi/ARBs |  |  |  |  |
| Received | 0.43 | 0.02 | 1.25 | 1.01 |
| Contraindicated/ not applicable | 0.11 | -0.03 | 2.15 | 0.86 |
| Statins |  |  |  |  |
| Received | 0.60 | 0.03 | 0.51 | 0.96 |
| Contraindicated/ not applicable | 0.04 | -0.01 | 1.37 | 0.94 |
| **In-hospital procedures** |  |  |  |  |
| Coronary angiography |  |  |  |  |
| Received | 0.14 | 0.002 | 0.97 | 1.00 |
| Contraindicated/ not applicable | -0.04 | -0.01 | 0.84 | 0.98 |
| Coronary intervention (PCI/CABG) |  |  |  |  |
| Received | 0.07 | -0.01 | 1.04 | 0.99 |
| Contraindicated/ not applicable | -0.02 | -0.001 | 0.93 | 1.00 |

**Abbreviations:** ACE, angiotensin converting enzyme; ARB, angiotensin receptor blocker; CABG, coronary artery bypass graft; CHD, coronary heart disease; COPD, chronic obstructive pulmonary disease; IMD, Index of multiple deprivation; PCI, percutaneous coronary intervention; ref, reference category.

**Table 13.** Balance check parameters using standardized differences and variance ratios (imputation dataset 10)

|  | **Standardized differences** | | **Variance ratios** | |
| --- | --- | --- | --- | --- |
| **Data** | **Raw** | **Weighted** | **Raw** | **Weighted** |
| Age |  |  |  |  |
| Below 55 | ref | ref | ref | ref |
| 55-65 | 0.03 | 0.02 | 1.06 | 1.04 |
| 66-75 | 0.07 | -0.01 | 1.09 | 0.99 |
| 76-85 | 0.02 | -0.01 | 1.02 | 0.99 |
| Above 85 | -0.09 | -0.01 | 0.86 | 0.99 |
| Male | 0.02 | 0.02 | 1.00 | 1.00 |
| Deprivation (IMD) |  |  |  |  |
| Least deprived (1) | ref | ref | ref | ref |
| 2 | 0.01 | -0.003 | 1.02 | 1.00 |
| 3 | 0.03 | -0.01 | 1.04 | 0.98 |
| 4 | -0.02 | -0.01 | 0.97 | 0.99 |
| Most deprived (5) | 0.004 | 0.01 | 1.01 | 1.02 |
| Year of admission |  |  |  |  |
| 2007 | ref | ref | ref | ref |
| 2008 | 0.07 | 0.02 | 1.11 | 1.02 |
| 2009 | -0.01 | 0.01 | 0.98 | 1.01 |
| 2010 | -0.03 | -0.01 | 0.94 | 0.99 |
| 2011 | -0.05 | -0.01 | 0.90 | 0.97 |
| 2012 | -0.06 | -0.01 | 0.83 | 0.97 |
| 2013 | -0.06 | -0.01 | 0.72 | 0.92 |
| **Cardiovascular history** |  |  |  |  |
| Cerebrovascular disease | -0.003 | -0.01 | 0.99 | 0.96 |
| Peripheral vascular disease | 0.06 | 0.01 | 1.37 | 1.07 |
| **Cardiovascular risk factors** |  |  |  |  |
| Diabetes | 0.03 | 0.02 | 1.07 | 1.04 |
| Hypercholesterolaemia | 0.05 | -0.01 | 1.07 | 0.99 |
| Hypertension | 0.05 | -0.01 | 1.02 | 1.00 |
| Current or ex-smoker | 0.10 | 0.01 | 0.97 | 1.00 |
| Asthma or COPD | 0.08 | -0.002 | 1.06 | 1.00 |
| Family history of CHD | 0.08 | -0.02 | 1.12 | 0.98 |
| **Presenting characteristics** |  |  |  |  |
| Heart rate >110 bpm | -0.06 | -0.02 | 0.85 | 0.95 |
| Creatinine >200 (μmol/l) | -0.05 | -0.01 | 0.76 | 0.95 |
| Peak troponin | 0.02 | 0.02 | 0.94 | 0.95 |
| Cardiac arrest | 0.03 | -0.01 | 1.27 | 0.93 |
| **Electrocardiographic characteristics** |  |  |  |  |
| ST-segment deviation | 0.04 | -0.01 | 1.01 | 1.00 |
| Care by cardiologist | 0.004 | -0.02 | 1.00 | 1.01 |
| **Medication at discharge** |  |  |  |  |
| Aspirin |  |  |  |  |
| Received | 0.46 | 0.03 | 0.44 | 0.93 |
| Contraindicated/ not applicable | 0.02 | 0.003 | 1.10 | 1.01 |
| P2Y_12_ inhibitors |  |  |  |  |
| Received | 0.55 | 0.03 | 0.64 | 0.97 |
| Contraindicated/ not applicable | 0.06 | -0.01 | 1.37 | 0.95 |
| ACEi/ARBs |  |  |  |  |
| Received | 0.44 | 0.01 | 1.25 | 1.00 |
| Contraindicated/ not applicable | 0.12 | 0.02 | 2.34 | 1.13 |
| Statins |  |  |  |  |
| Received | 0.63 | 0.03 | 0.50 | 0.96 |
| Contraindicated/ not applicable | 0.04 | 0.01 | 1.38 | 1.10 |
| **In-hospital procedures** |  |  |  |  |
| Coronary angiography |  |  |  |  |
| Received | 0.15 | -0.001 | 0.96 | 1.00 |
| Contraindicated/ not applicable | -0.03 | -0.01 | 0.89 | 0.95 |
| Coronary intervention (PCI/CABG) |  |  |  |  |
| Received | 0.07 | -0.01 | 1.04 | 1.00 |
| Contraindicated/ not applicable | -0.002 | 0.01 | 0.99 | 1.02 |

**Abbreviations:** ACE, angiotensin converting enzyme; ARB, angiotensin receptor blocker; CABG, coronary artery bypass graft; CHD, coronary heart disease; COPD, chronic obstructive pulmonary disease; IMD, Index of multiple deprivation; PCI, percutaneous coronary intervention; ref, reference category.

**Table 14.** Over-identification test results for each of the imputed datasets used for the analysis

| **Imputation** | **P-value for AMI analysis** | **P-value for STEMI analysis** | **P-value for NSTEMI analysis** |
| --- | --- | --- | --- |
| 1 | 0.20 | 0.27 | 0.47 |
| 2 | 0.07 | 0.87 | 0.10 |
| 3 | 0.89 | 0.48 | 0.35 |
| 4 | 0.39 | 0.87 | 0.17 |
| 5 | 0.55 | 0.36 | 0.70 |
| 6 | 0.07 | 0.64 | 0.10 |
| 7 | 0.25 | 0.87 | 0.05 |
| 8 | 0.29 | 0.89 | 0.60 |
| 9 | 0.36 | 0.88 | 0.22 |
| 10 | 0.28 | 0.53 | 0.71 |

**Abbreviations:** AMI, acute myocardial infarction; NSTEMI, non ST-segment elevation myocardial infarction; STEMI, ST-segment elevation myocardial infarction.

**Figure 6:** Area under ROC curve for the propensity scoring model


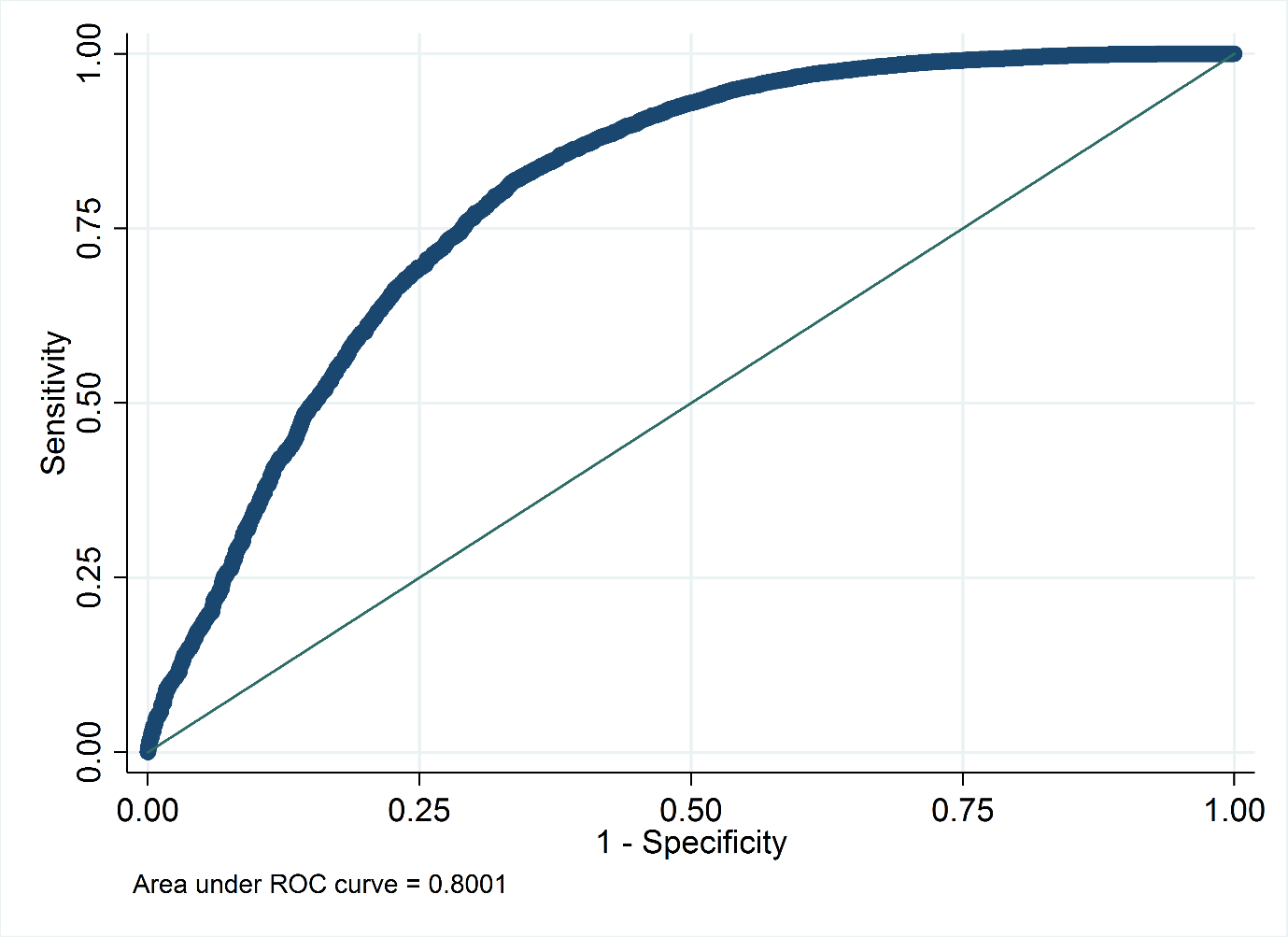


**Figure 7:** Adjusted survival (Kaplan-Meier estimates) among patients prescribed β blockers at discharge and those not prescribed (N= 179,810)


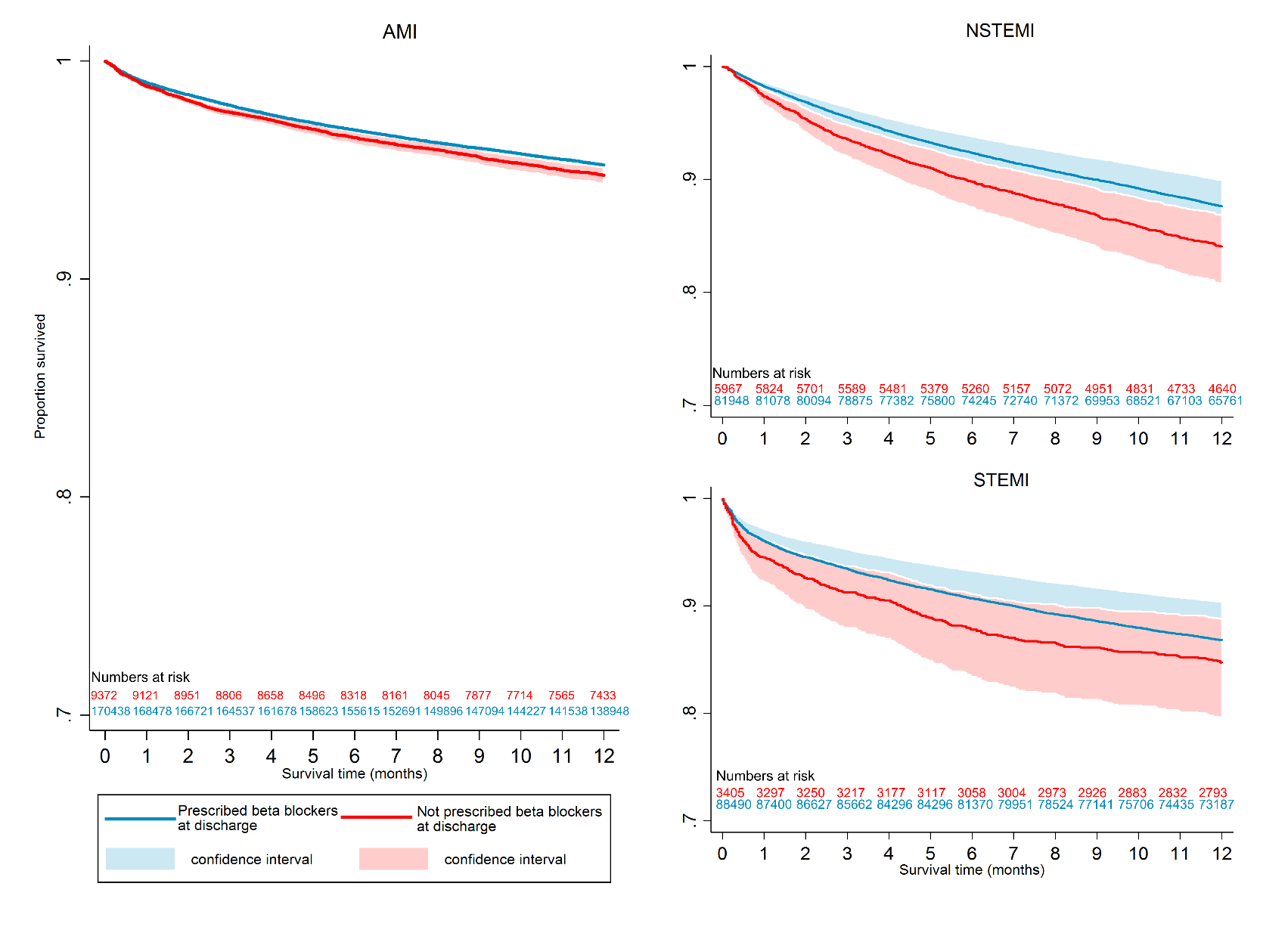


**Section 3: Instrumental variable analysis**

In order to mitigate potential selection bias introduced by measured or unmeasured confounding in observational data, an instrumental variable analysis was performed.([5](#_ENREF_5)) The use of instrumental variable analysis allowed us to determine the association between the use of β blockers and survival, whilst using an instrument to behave like a natural randomisation of patients to hospitals that differ in their likelihood of receiving treatment, and to provide closer approximations to the average population effects from randomized clinical trials.

We used hospital rates of prescription of guideline-indicated treatments (aspirin, P2Y_12_ inhibitors, β blockers, statins and angiotensin converting enzyme inhibitors (ACEi) or angiotensin receptor blockers (ARB)) as the instrumental variable. Our choice was informed by clinical knowledge and past literature which have found that “physician prescribing preference” is a good instrument for investigating drug effectiveness when using instrumental variable analysis.([5](#_ENREF_5)) We hypothesised that hospital prescribing rates of discharge medications as an instrument would behave similarly to physician prescribing preferences. Validity of the instrument was confirmed by checking that it was correlated with receipt of β blockers at discharge, was independent of other patient characteristics and was independent of patient outcomes. To test the first assumption we used a multilevel logistic regression model to predict β blocker use at discharge as a function of hospital prescribing rates, for assumption two we compared patient characteristics across quintiles of the instrument and for the third assumption we regressed mortality on the instrumental variable after adjusting for β blocker use at discharge and other patient characteristics. At the time of analysis there were no available packages (to our best knowledge) which allowed the analysis of instrumental variables for time to event data. Previous studies have used logistic regression modelling as a proxy for time to event data with a binary outcome for mortality. However, this leads to survivorship bias because it does not consider follow-up time. To mitigate survivorship bias we used Poisson regression modelling with an offset for the log of survival time to post discharge up to 180 days as in our previous work.([6](#_ENREF_6)) This provided a better approximation to the survival modelling framework of our analysis modelling strategy.

We found that the instrument variable was a good predictor of use of β blockers, was well balanced across patient characteristics (Table 15) and was independent of the patient outcomes (30 day mortality; OR 1.11, 95% CI 0.38 to 3.23, P=0.847, 6 months mortality; 1.45, 0.68 to 3.07, P=0.337 and 1 year mortality; 1.25, 0.63 to 2.46, P=0.522) to therefore meet the required assumptions as a valid instrument.

**Table 15.** Patient characteristics and mortality according to quintiles of hospital prescribing rates of five drugs at discharge^*^

|  | **Quintile of hospital prescribing rates of five drugs at discharge** | | | | |
| --- | --- | --- | --- | --- | --- |
| **Variable^a^** | **1**  **N=28,870** | **2**  **N=35,981** | **3**  **N=33,775** | **4**  **N=39,021** | **5**  **N=42,163** |
| Age, mean (SD), years | 65.0 (13.7) | 64.4 (13.7) | 64.0 (13.8) | 64.1 (13.5) | 63.7 (13.6) |
| Male | 19,767 (68.5) | 25,166 (69.9) | 23,258 (68.9) | 27,641 (70.8) | 30,029 (71.2) |
| Deprivation (IMD score), median (IQR) | 17.7 (10.6 -30.1) | 16.5 (9.5-28.4) | 20.1 (11.6-35.1) | 17.0 (9.9-30.2) | 17.5 (9.7-32.4) |
| **Year of admission** |  |  |  |  |  |
| 2007 | 5,090 (17.6) | 5,398 (15.0) | 4,607 (13.6) | 5,228 (13.4) | 4,703 (11.2) |
| 2008 | 4,905 (17.0) | 5,026 (14.0) | 4,675 (13.8) | 5,956 (15.3) | 5,556 (13.2) |
| 2009 | 5,195 (18.0) | 5,242 (14.6) | 5,086 (15.1) | 6,264 (16.1) | 6,911 (16.4) |
| 2010 | 4,522 (15.7) | 6,148 (17.1) | 5,534 (16.4) | 6,277 (16.1) | 7,425 (17.6) |
| 2011 | 4,166 (14.4) | 6,064 (16.9) | 5,935 (17.6) | 6,551 (16.8) | 7,487 (17.8) |
| 2012 | 3,973 (13.8) | 6,114 (17.0) | 5,970 (17.7) | 6,577 (16.9) | 7,609 (18.1) |
| 2013 | 1,019 (3.5) | 1,989 (5.5) | 1,968 (5.8) | 2,168 (5.6) | 2,472 (5.9) |
| **Cardiovascular history** |  |  |  |  |  |
| Cerebrovascular disease | 1,272 (4.4) | 1,271 (3.5) | 1,542 (4.6) | 1,374 (3.5) | 1,456 (3.5) |
| Peripheral vascular disease | 476 (1.7) | 644 (1.8) | 818 (2.4) | 668 (1.7) | 805 (1.9) |
| **Cardiovascular risk factors** |  |  |  |  |  |
| Diabetes | 3,266 (11.3) | 4,253 (11.8) | 3,985 (11.8) | 4,614 (11.8) | 4,637 (11.0) |
| Chronic renal failure | 505 (1.8) | 537 (1.5) | 631 (1.9) | 578 (1.5) | 652 (1.6) |
| Hypercholesterolaemia | 6,371 (22.1) | 7,955 (22.1) | 8,122 (24.1) | 8,577 (22.0) | 10,925 (25.9) |
| Hypertension | 10,012 (34.7) | 11,955 (33.2) | 11,754 (34.8) | 12,392 (31.8) | 14,231 (33.8) |
| Current or Ex-smoker | 18,555 (64.3) | 23,175 (64.4) | 22,306 (66.0) | 25,401 (65.1) | 27,899 (66.2) |
| Asthma or COPD | 3,348 (11.6) | 3,698 (10.3) | 3,922 (11.6) | 3,930 (10.1) | 4,442 (10.5) |
| Family history of CHD | 9,052 (31.4) | 9,850 (27.4) | 10,211 (30.2) | 10,946 (28.1) | 13,140 (31.2) |
| **Presenting characteristics** |  |  |  |  |  |
| Systolic blood pressure, mmHg , mean (SD) | 141.3 (27.2) | 140.4 (27.5) | 140.6 (27.7) | 139.1 (27.4) | 138.1 (27.0) |
| Heart rate, (beat/min), median (IQR) | 77.0 (66.0-90.0) | 78.0 (67.0-90.0) | 77.0 (66.0-90.0) | 76.0 (66.0-89.0) | 76.0 (65.0-89.0) |
| Creatinine, (mg/dL), median (IQR) | 86.0 (73.0-101.0) | 86.0 (73.0-101.0) | 84.0 (71.0-99.0) | 85.0 (72.0-99.0) | 85.0 (73.0-100.0) |
| Peak troponin^§^, median (IQR) | 3.3 (0.4-45.3) | 3.6 (0.4-50.0) | 4.3 (0.5-44.7) | 5.8 (0.7-50.0) | 3.7 (0.5-45.7) |
| Cardiac arrest | 860 (3.0) | 1,222 (3.4) | 1,106 (3.3) | 1,535 (3.9) | 1,900 (4.5) |
| **Electrocardiographic characteristics** |  |  |  |  |  |
| No acute changes | 3,862 (13.4) | 3,981 (11.1) | 4,177 (12.4) | 3,807 (9.8) | 3,401 (8.1) |
| ST-segment elevation | 11,879 (41.2) | 17,137 (47.6) | 16,105 (47.7) | 20,617 (52.8) | 23,281 (55.2) |
| Left bundle branch block | 612 (2.1) | 860 (2.4) | 704 (2.1) | 852 (2.2) | 828 (2.0) |
| ST segment depression | 4,185 (14.5) | 4,429 (12.3) | 3,797 (11.2) | 4,373 (11.2) | 4,534 (10.8) |
| T wave changes only | 5,146 (17.8) | 5,761 (16.0) | 4,951 (14.7) | 6,105 (15.7) | 6,516 (15.5) |
| Other acute abnormality | 3,186 (11.0) | 3,813 (10.6) | 4,041 (12.0) | 3,267 (8.4) | 3,603 (8.6) |
| **Grace risk score** |  |  |  |  |  |
| Lowest (≤70) | 3,195 (11.1) | 3,749 (10.4) | 3,402 (10.1) | 3,321 (8.5) | 3,659 (8.7) |
| Low (71-87) | 4,235 (14.7) | 5,207 (14.5) | 4,693 (13.9) | 5,040 (12.9) | 5,684 (13.5) |
| Intermediate to high (>88) | 21,440 (74.3) | 27,025 (75.1) | 25,680 (76.0) | 30,660 (78.6) | 32,820 (77.8) |
| **Index event** |  |  |  |  |  |
| NSTEMI | 16,435 (56.9) | 18,285 (50.8) | 17,038 (50.5) | 17,661 (45.3) | 18,496 (43.9) |
| **Medication at discharge^b^** |  |  |  |  |  |
| Aspirin | 27,309 (97.0) | 34,402 (98.0) | 32,715 (99.1) | 37,869 (99.2) | 40,965 (99.4) |
| P2Y_12_ inhibitors | 25,535 (91.7) | 32,368 (94.1) | 30,911 (96.5) | 36,063 (96.8) | 39,094 (97.3) |
| ACEi/ARBs | 23,618 (86.2) | 31,322 (92.3) | 30,035 (94.7) | 34,463 (95.5) | 38,255 (97.4) |
| Statin | 27,060 (94.9) | 34,206 (96.7) | 32,634 (98.1) | 37,704 (98.6) | 40,807 (99.0) |
| β blockers | 25,835 (89.5) | 33,650 (93.5) | 32,262 (95.5) | 37,680 (96.6) | 41,063 (97.4) |
| **In-hospital procedures^b^** |  |  |  |  |  |
| Coronary angiography | 19,751 (71.1) | 24,455 (69.8) | 24,055 (74.3) | 23,628 (63.3) | 28,978 (71.6) |
| Coronary intervention | 14,846 (54.6) | 18,610 (54.3) | 18,895 (59.3) | 18,071 (49.9) | 23,606 (59.7) |
| **Rehabilitation^b^** |  |  |  |  |  |
| Enrolment into cardiac rehabilitation | 24,665 (89.2) | 32,398 (94.1) | 29,885 (92.7) | 34,571 (92.2) | 38,444 (96.3) |

**Abbreviations:** ACE, angiotensin converting enzyme inhibitor; ARB, angiotensin receptor blocker; CABG, coronary artery bypass graft; CHD, coronary heart disease; COPD, chronic obstructive pulmonary disease; PCI, percutaneous coronary intervention; IMD, Index of multiple deprivation; IQR, interquartile range; SD, standard deviation; § peak troponin was truncated at 50; *****Five discharge drugs (aspirin, P2Y_12_ inhibitors, β blockers, statins and angiotensin converting enzyme inhibitors (ACEi) or angiotensin receptor blockers (ARB)).

**References**

1. Cattle B, Baxter P, Greenwood D, Gale C, West R. Multiple imputation for completion of a national clinical audit dataset. Stat Med 2011;30:2736-2753.

2. Imai K, Ratkovic M. Covariate balancing propensity score. Journal of the Royal Statistical Society: Series B (Statistical Methodology) 2014;76:243-263.

3. Crump RK, Hotz VJ, Imbens GW, Mitnik OA. Dealing with limited overlap in estimation of average treatment effects. Biometrika 2009:asn055.

4. Austin PC. The use of propensity score methods with survival or time‐to‐event outcomes: reporting measures of effect similar to those used in randomized experiments. Stat Med 2014;33:1242-1258.

5. Rassen JA, Brookhart MA, Glynn RJ, Mittleman MA, Schneeweiss S. Instrumental variables II: instrumental variable application—in 25 variations, the physician prescribing preference generally was strong and reduced covariate imbalance. Journal of clinical epidemiology 2009;62:1233-1241.

6. Hall M, Dondo TB, Yan AT et al. Association of clinical factors and therapeutic strategies with improvements in survival following non–ST-elevation myocardial infarction, 2003-2013. JAMA 2016;316:1073-1082.
